# Supplementary material for: Patient flow within UK emergency departments: a systematic review of the use of computer simulation modelling methods
Source: BMJ Open. 2017 May 9;7(5):e015007. doi: 10.1136/bmjopen-2016-015007 (PMC5566625; doi:10.1136/bmjopen-2016-015007)
Supplement: Supplementary data [file bmjopen-2016-015007supp001.pdf]

APPENDIX 1

MEDLINE search strategy

----

1 exp Computer Simulation/ (175146)

2 models, statistical/ (75054)

3 ((computer* or distributed or hybrid) adj6 (model* or simulation*)).mp. (184347)

4 ((flow or simulation) adj6 model$).mp. (41697)

5 (system adj3 model$).mp. (50666)

6 (discrete adj2 event).mp. (783)

7 (monte adj2 carlo).mp. (40800)

8 markov.mp. (17983)

9 (statechart$ or stochastic or system dynamics).mp. (32076)

10 (agent adj2 based).mp. (3106)

11 or/1-10 (391259)

12 workflows/ or crowding/ or exp waiting lists/ (13340)

13 time to treatment/ or triage/ or patient admission/ or length of stay/ (92230)

14 exp critical pathways/ or exp "Process Assessment (Health care)"/ (8528)

15 exp patient care planning/ or exp patient care/ or exp "continuity of patient care"/ (679418)

16 ((clinical or critical or patient*) adj2 (path or paths or pathways)).tw. (5722)

17 (patient$ adj3 (flow or flows or flowing or throughput)).tw. (11371)

18 (care adj3 (step or steps or stage$)).tw. (2424)

19 (process adj2 care).tw. (4165)

20 (workflow* or crowding* or waiting list* or waiting time*).tw. (31590)

21 or/12-20 (741748)

22 emergency medical services/ or emergency medical service communication systems/ or emergency service, hospital/ or exp ambulances/ (86523)

23 ((emergency or emergencies or unplanned or unscheduled) adj3 (care or admission$ or department$ or ward or wards or service$ or unit or units or room or rooms)).tw. (95179)

24 (A&E or "accident and emergency").tw. (18002)

25 (minor injur* adj2 (unit* or servic* or ward* or hospital* or department*)).ti,ab. (137)

26 22 or 23 or 24 or 25 (154509)

27 11 and 21 and 26 (437)
