# Supplementary material for: Patient flow within UK emergency departments: a systematic review of the use of computer simulation modelling methods
Source: BMJ Open. 2017 May 9;7(5):e015007. doi: 10.1136/bmjopen-2016-015007 (PMC5566625; doi:10.1136/bmjopen-2016-015007)
Supplement: Author's manuscript [file bmjopen-2016-015007.draft_revisions.pdf]

BMJ Open

A systematic review of the use of computer simulation modelling of patient flow within UK emergency departments

Journal:	*BMJ Open*
Manuscript ID	bmjopen-2016-015007
Article Type:	Research
Date Submitted by the Author:	02-Nov-2016

Complete List of Authors:	Mohiuddin, Syed; University Hospitals Bristol NHS Foundation Trust, NIHR CLAHRC West; University of Bristol, School of Social and Community Medicine<br>Busby, John; Queen's University Belfast, School of Medicine<br>Savovic, Jelena; University Hospitals Bristol NHS Foundation Trust, NIHR CLAHRC West; University of Bristol, School of Social and Community Medicine<br>Richards, Alison; University Hospitals Bristol NHS Foundation Trust, NIHR CLAHRC West; University of Bristol, School of Social and Community Medicine<br>Northstone, Kate; University Hospitals Bristol NHS Foundation Trust, NIHR CLAHRC West; University of Bristol, School of Social and Community Medicine<br>Hollingworth, William; University Hospitals Bristol NHS Foundation Trust, NIHR CLAHRC West; University of Bristol, School of Social and Community Medicine<br>Donovan, Jenny; University Hospitals Bristol NHS Foundation Trust, NIHR CLAHRC West; University of Bristol, School of Social and Community Medicine<br>Vasilakis, C; University of Bath, School of Management

**Primary Subject Heading**:	Health services research
Secondary Subject Heading:	Emergency medicine, Communication, Health policy, Medical management, Research methods
Keywords:	Systematic review, Emergency care, Overcrowding, Computer simulation, Patient flow, Waiting times

SCHOLARONE™
Manuscripts

A systematic review of the use of computer simulation modelling of patient flow within UK emergency departments

Syed Mohiuddin,[1,2*] John Busby,[3] Jelena Savović,[1,2] Alison Richards,[1,2] Kate Northstone,[1,2]
William Hollingworth,[1,2] Jenny L. Donovan,[1,2] Christos Vasilakis[4]

[1]NIHR CLAHRC West, University Hospitals Bristol NHS Foundation Trust, Bristol, UK
[2]School of Social and Community Medicine, University of Bristol, Bristol, UK
[3]School of Medicine, Queen's University Belfast, Belfast, UK
[4]School of Management, University of Bath, Bath, UK

[*]corresponding author:

Address: Whitefriars (9th Floor), Bristol BS1 2NT, UK

E-mail: syed.mohiuddin@bristol.ac.uk

Telephone: +44 (0)117 342 1267

Keywords:

Systematic review; Emergency care; Overcrowding; Computer simulation; Patient flow; Waiting times

Word count:

3,996

For peer review only

ABSTRACT

Objectives: Overcrowding in the emergency department (ED) is common in the UK as in other countries worldwide. Computer simulation is one approach used for understanding the causes of ED overcrowding and assessing the likely impact of changes to the delivery of emergency care. However, little is known about the usefulness of computer simulation for analysis of ED patient flow. We undertook a systematic review to investigate the different computer simulation methods and their contribution for analysis of patient flow within EDs in the UK.

Methods: We searched eight bibliographic databases (MEDLINE; EMBASE; COCHRANE; WEB OF SCIENCE; CINAHL; INSPEC; MATHSCINET; and ACM DIGITAL LIBRARY) from date of inception until 31 March 2016. Studies were included if they used a computer simulation method to capture patient progression within the ED of an established UK NHS hospital. Studies were summarised in terms of simulation method, key assumptions, input and output data, conclusions drawn, and implementation of results.

Results: Twenty one studies met the inclusion criteria. Of these, 19 used discrete event simulation and 2 used system dynamics models. The purpose of many of these studies (n=16; 76%) centred on service redesign. Seven studies (33%) provided no details about the ED being investigated. Most studies (n=18; 86%) used specific hospital models of ED patient flow. Overall, the reporting of underlying modelling assumptions was poor. Nineteen studies (90%) considered patient waiting or throughput times as the key outcome measure. Twelve studies (57%) reported some involvement of stakeholders in the simulation study. However, only 3 studies (14%) reported on the implementation of changes supported by the simulation.

Conclusions: We found that computer simulation can provide a means to pre-test changes to ED care delivery before implementation in a safe and efficient manner. However, there are some methodological, data, stakeholder, implementation, and reporting issues, which must be addressed by future studies.

For peer review only - http://bmjopen.bmj.com/site/about/guidelines.xhtml

Strengths and limitations of this study

- We systematically reviewed the peer-reviewed literature to investigate the contribution of various computer simulation methods for analysis of patient flow within EDs in the UK.
- We searched eight bibliographic databases to identify the relevant studies. Further to the electronic search, we used hand-searching to review the backward and forward citations of all included studies.
- We highlighted a number of methodological, data, stakeholder, implementation, and reporting related issues associated with current studies.
- We included studies which were conducted under the jurisdiction of UK NHS only.
- We did not assess the quality of the identified simulation models since we are not aware of any guidelines to carry out this type of quality assessment.
- We recommend the adoption of reporting guidelines by academic journals and conference proceedings, and more persistent exploitation of innovative models of engagement and knowledge mobilisation between academics and healthcare professionals such as the Researchers in Residence.

For peer review only

INTRODUCTION

An emergency department (ED), also known as accident & emergency department (A&E), provides acute care for patients who attend hospital without prior appointment. The EDs of most hospitals customarily operate 24 hours a day, 7 days a week. Nevertheless, overcrowding in EDs is an increasing problem in countries around the world, and especially so in the United Kingdom (UK).[1 2] ED overcrowding has been shown to have many adverse consequences such as increased medical errors,[3] decreased quality of care and subsequently poor patient outcomes,[4] increased workload,[1] frustration among ED staff,[4 5] ambulance diversions,[6] increased patient dissatisfaction,[5] prolonged patient waiting times,[7] and increased cost of care.[8] Furthermore, some less severely ill patients may leave without being seen by a physician, only to return later with a more complicated condition.[8]

In the UK, there is an enormous pressure from public and government to alleviate overcrowding and long waiting times experienced in ED.[1] The Department of Health set a target standard for acute hospitals in the National Health Service (NHS) that at least 95% of patients attending an A&E department must be seen, treated, admitted, or discharged in under four hours.[7] This 4-hour target standard was initially set at 98% in 2004, but later reduced to 95% in 2010. Beyond target setting, it has been argued that ED overcrowding can be improved by lean healthcare thinking with a focus on improving patient flow.[1 9]

Over recent decades, computer simulation and other modelling methods have been used to analyse ED patient flow and resource capacity planning.[10-14] In essence, a computer simulation model is a simplified representation of reality used to aid the understanding of the key relationships and dynamics in the care process, and to evaluate the likely impact of changes before implementation. Typically, a simulation model is based on the notion that each simulated individual is tracked through the care process; the population effect is then estimated from the sum of the individual effects.[15]

The precise way in which a simulation model works depends on the type of simulation method used. Generally, simulation models can be categorised as static or dynamic, as stochastic or deterministic, and as discrete-time or continuous-time.[16] A static simulation represents a process at a particular point in time, whereas a dynamic simulation represents a process as it evolves over time. A simulation model in which at least one input parameter is a random variable is said to be stochastic, whereas a simulation model having no random variables is said to be deterministic. A discrete-time model is one in which the state variables change instantaneously at discrete points in time. In contrast, a continuous-time model is one in which the state variables change continuously with respect to time. The advancement of computer technology has undoubtedly supported the utilisation of more sophisticated simulation methods for modelling healthcare processes. Today, for

For peer review only - http://bmjopen.bmj.com/site/about/guidelines.xhtml

example, computer simulation is also capable of providing an insight into the workings of a system through visual animation.

Various types of simulation exist, including discrete event simulation (DES) and system dynamics (SD). DES is a widely used method, and can replicate the behaviour of complex healthcare systems over time. A DES model is a network of queues and activities (such as having a blood test, x-ray, and treatment). One of the major advantages of using a DES model is its flexibility to model complex scenarios at the individual level. Within a DES model, individuals move from one activity to another in sequential order at a particular point in time. Typically, the individuals enter a system and visit some of the activities (not necessarily only once) before leaving the system. The variables that govern the movement of modelled individuals (e.g. arrival rate, duration of treatment, etc.) can be random and thus readily capture the variation that is inherent in healthcare. As such, a DES model is considered particularly suitable for modelling queuing systems. This simulation method is able to incorporate life histories and complex scenarios at the individual level to influence the care pathway taken and the time between events, but specialist analytical knowledge is required typically to achieve a greater flexibility.[17]

Another widely used computer simulation method is system dynamics, which is used to understand the behaviour of complex healthcare systems over time through capturing aggregate (instead of individual) flows of patients. An SD model is essentially collection of stocks and flows between them. Stocks are basic stores of quantities over time, e.g. number of patients with a disease or in a particular part of a hospital department. Flows define the movement of objects between different stocks over time. Unlike DES, system dynamics models cannot include random variables and thus input parameters are given as simple rates. For these reasons, SD is considered not the ideal method of choice for modelling a closely focussed system that involves resource-constrained queueing networks, such as an ED.[10] In a situation like this, DES should rather be the method of choice to model high level of distinct detail.[18]

Increasing interest in this area is reflected in the number of computer simulation studies of ED patient flow and resource capacity planning that have been published over recent decades. However, little is known about the usefulness of different computer simulation methods for analysis of any changes to the delivery of emergency care. We, therefore, systematically investigated the peer-reviewed literature on the use of computer simulation modelling of patient flow within EDs in the UK. Our specific objectives were as follows:

i. to investigate the contribution that computer simulation studies make to our understanding of the problem of ED overcrowding;
ii. to identify the methodology used to conduct patient flow simulation in terms of key assumptions, systems requirements, and input and output data;

For peer review only - http://bmjopen.bmj.com/site/about/guidelines.xhtml

iii. to assess the usefulness of each simulation method for service redesign and evaluating the likely impact of changes related to the delivery of emergency care;

iv. to report on differences in conclusions about ED performance with different simulation modelling methods; and

v. to identify studies that explicitly aimed to meet the pre-specified needs of stakeholders.

METHODS

We conducted a systematic review of the peer-reviewed literature to identify computer simulation studies of patient flow within hospital EDs in the UK. This review complies with the PRISMA checklist (www.prisma-statement.org). We produced a review protocol (available from the corresponding author on request) and set out the process to address our specific objectives.

Search strategy

We retrieved relevant studies from the following bibliographic databases: MEDLINE; EMBASE; COCHRANE; WEB OF SCIENCE; CINAHL; INSPEC; MATHSCINET; and ACM DIGITAL LIBRARY. These databases were searched from the date of their inception until 31 March 2016. A search strategy was designed to explore three main domains of knowledge associated with the area of our interest: computer simulation; patient flow; and emergency care. We included a wide range of search terms, both keywords and medical subject headings, associated with each domain of knowledge. We created the search strategies for all databases. However, we first developed the search strategy for MEDLINE (Appendix 1) since it is known to allow a rich taxonomy of subjects and rubrics, and then used the MEDLINE strategy as a template to define similar strategies for the other databases (available from the corresponding author on request).

Inclusion criteria

We identified studies as being eligible for inclusion if they:

i. were published in peer-reviewed journals or conference proceedings as full papers;

ii. were conducted within the ED of an established UK NHS hospital responsible for assessing and treating civilians in need of emergency care;

iii. captured the progress of patients through at least two activities of an ED care process; and

iv. used a computer simulation method such as discrete event simulation, system dynamics, agent based simulation, hybrid simulation, Monte Carlo simulation, distributed simulation, or stochastic modelling.

For peer review only - http://bmjopen.bmj.com/site/about/guidelines.xhtml

For peer review only

We excluded editorials, letters, commentaries, conference abstracts, notes, and books. We also excluded studies that used methods such as regression analysis, likelihood ratio test, time series analysis, generalised linear model, mathematical programming, optimisation methods, queuing theory, structural equation modelling, process mapping, problem structuring method, or risk analysis without combining it with a computer simulation method.

Selection of studies for full-text review

To identify the studies suitable for full-text review, two authors (SM and JB) independently screened the titles and abstracts of all the initially retrieved studies. The individual responses from each reviewer were stored in a common database. At this stage, a study was excluded if it was clearly irrelevant based on our inclusion criteria. In cases of discrepancy, we selected the studies for full-text review by consensus.

Appraisal of studies for inclusion

An electronic questionnaire was designed to appraise the studies selected for full-text review. The questionnaire included four key questions: (i) is the study a full paper published in a peer-reviewed journal or conference proceedings; (ii) is the study set within the UK NHS; (iii) is the study conducted within the ED of an established hospital; and (iv) does the study use a computer simulation model of emergency patient flow? A study with positive responses on these four questions was then included in the final review. SM and JB completed this process independently and resolved any discrepancies that arose by consensus. Further to the electronic search, SM and JB used hand-searching and reviewed the backward and forward citations of all studies included in the electronic search, to identify other potentially relevant studies.

Data extraction

An electronic data extraction form was created to retrieve information about a number of key aspects, including simulation methods, data sources, key assumptions, input and output data, conclusions drawn, and benefits of simulation outputs in practice. SM and JB independently recorded, collated, and extracted the necessary information. Any discrepancies were resolved by consensus.

RESULTS

We retrieved a total of 2,436 references from the eight databases: 437 from MEDLINE; 460 from EMBASE; 14 from COCHRANE; 253 from WEB OF SCIENCE; 65 from CINAHL; 1103 from INSPEC; 4

For peer review only - http://bmjopen.bmj.com/site/about/guidelines.xhtml

from MATHSCINET; and 100 from ACM DIGITAL LIBRARY. We removed 440 duplicate references, and then assessed the remaining 1,996 unique references by title and abstract screening. At this stage, we selected 159 of the 1,996 studies for full-text review. Nineteen of the 159 studies were included following full-text review. Two more studies were included from the backward and forward hand-searching of the 19 studies. A total of 21 studies[10-14 19-34] were included in the final review. Four studies[35-38] were excluded from the final review because the models used by these studies were identical to the models reported in other already included studies.[13 28 31 34] Figure 1 shows a summary of the study selection process.

<Figure 1>

Table 1 summarises the included studies, outlining publication type, simulation purpose, ED details, and patient flow description. The first study[31] was published in 2000 and the most recent study[19] was published in 2013. The maximum number of studies (n=4) published in any single year was in 2006 and 2011. Nine of the 21 included studies (43%) were published in conference proceedings. The highest number of studies (n=7) was published in proceedings of the Winter Simulation Conference, the second highest (n=5) was in the Emergency Medicine Journal, and the third highest (n=3) was in the Journal of the Operational Research Society. More than two thirds of the studies (n=16; 76%) did not provide the name of the hospital studied. All 21 studies described the underlying purpose of simulation; and in many cases (n=16; 76%), this centred on service redesign. Surprisingly, 7 studies[12 13 19 23 29 32 34] (33%) did not provide any details about the ED being investigated, while 5 studies[10 19 21 24 28] (24%) did not provide patient flow diagram.

<Table 1>

Table 2 provides summary of simulation methods, including simulation type, key assumptions, and use of software. The types of simulation varied only between two methods (DES and SD). DES modelling was used in 19 studies (90%), while SD was used in 2 studies (10%). All but one study[23] either explicitly or implicitly justified the choice of underlying modelling method used. The majority of studies (n=18; 86%) used specific hospital models of ED patient flow. The reporting of modelling assumptions was poor overall. For example, as many as 12 studies[10 12 13 19 21-23 27 29 30 32 33] (57%) did not provide any details about simulation duration, warmup period, and run number. Only 5 studies[11 20 25 28 31] (24%) specified the number of simulation runs and 3 studies[25 28 34] (14%) specified the simulation warmup period. Simulation duration ranged between 24 hours[25 31] and 52 weeks.[24 28]

For peer review only - http://bmjopen.bmj.com/site/about/guidelines.xhtml

Almost 50% (n=10) of the studies used Simul8 software (www.simul8.com) for running the model. Two studies[22 32] (10%) did not provide any details about the use of software.

<Table 2>

Table 3 provides detail of simulation inputs and outputs. The identified models were populated from three sources of data: primary (i.e. collected within the hospital being studied), secondary (i.e. collected in another setting), and expert opinion. One study[19] did not describe the source of data for any of the model inputs. Three studies[13 24 29] described the source of patient arrival rates, but not the sources of activity duration, activity progression, and use of resources. Eight studies (38%) stated explicitly that they used expert opinion to populate some of the model inputs. The proportion of studies that used primary data was reasonably high (Table 3). In particular, 95% (n=20) of the studies used primary data for patient arrival rates, 67% (n=14) for activity duration, 62% (n=13) for activity progression, and 52% (n=11) for resource inputs.

The most common changes considered in the simulation studies were ED patient flow (e.g. changes in the triage system for arriving patients[28]) and resource capacity planning (e.g. changes in the number of cubicles[28]). However, one third of the studies[11 13 19 22 24 25 29] (n=7) did not provide any details about the changes considered by the simulation. The majority of studies (n=19; 81%) considered patient waiting times (i.e. time from arrival to discharge, admission, or transfer) as the key outcome measure. In particular, 11 studies[10 11 13 19 20 23 25 27-29 32] considered patient waiting times alone, 7 studies[12 21 22 26 30 33 34] considered patient waiting times and resources used, and the other study[31] considered patient waiting times, resources used, and elective cancellations. Two other outcome measures considered were resources used[24] and bed occupancy.[14]

Only 12 studies (57%) reported some involvement of stakeholders in the simulation study, mainly when deciding the study questions or specifying the model structure. However, in the study conducted by Mould et al.,[34] stakeholders were involved in deciding the study questions, specifying the model structure, and implementing the model outputs. More than 80% (n=17) of the studies carried out some form of validation, mainly face and/or data-led validation. In face validation, project team members, potential users and other stakeholders subjectively compare model and real-life behaviours to judge whether the model and its results are reasonable at “face value”.[39] Data-led validation involves the comparing of model output with “real world” data and may also include a sensitivity analysis to determine the effect of varying the model’s inputs on its output performance.[40]

<Table 3>

For peer review only - http://bmjopen.bmj.com/site/about/guidelines.xhtml

Table 4 describes simulation results, summarising conclusions in terms of whether the changes considered (e.g. increase in staffing numbers) were supported by the simulation, whether the changes supported were implemented in practice (e.g. staffing increased), and barriers to conducting the simulation (e.g. data issues) and implementing the changes supported (e.g. poor clinician buy-in and credibility). Two thirds of the studies (n=14; 67%) provided some discussion on the usefulness of simulation for analysis of changes to the delivery of emergency care (Table 4): 6 studies supported the proposed changes, 1 study opposed the proposed changes, and 7 studies recommended differential changes. Only a small number of studies[12 32 34] (n=3; 14%) reported that the proposed changes supported by the simulations were implemented. For example, Maull et al.[32] estimated the impact of introducing a 'see and treat' strategy to reduce patient waiting times in the ED. After implementation, the observed reduction in breaches of the 4-hour waiting time target closely mirrored the simulation model predictions.

We identified a broad range of challenges, including oversimplified assumptions[20 23 31 33] and model structure,[14 23] system complexity,[11 14 28 29 32] poor data quality,[12 23 27 32 34] high expectations,[22] short-timescale,[31] poor stakeholder engagement,[12] limited specialist analytical skills,[34] model runtime,[11 22] generalisability,[14 26] and impact of simulation;[34] 6 studies[10 19 21 24 25 30] (29%) did not describe any emergent issues.

<Table 4>

DISCUSSION

This review has shown that computer simulation has been used to analyse ED patient flow and resource capacity planning to the delivery of emergency care. The most common types of computer simulation used were DES (n=19; 90%) and SD (n=2; 10%). All but one study[23] provided either explicit or implicit justification for the choice of modelling method used. However, the use of computer simulation of patient flow within EDs in the UK does not appear to have increased in recent years as may have been expected. This could be a reflection of the relatively limited availability of funding for research in this area compared to funding for health technology assessment. There are also a limited number of research groups with the analytical skills required to develop technically complex simulation models for the analysis of service redesign.

Identified studies varied in the style and quality of reporting; but assumptions used in the analyses were not always transparently reported. The opaque reporting of key assumptions prevents decision-makers from appraising the quality of evidence from simulation experiments. Although there is a set of guidelines for reporting inputs and outputs from DES modelling,[41] this has

For peer review only - http://bmjopen.bmj.com/site/about/guidelines.xhtml

not been widely adopted yet. Most of the studies (n=19; 90%) considered patient waiting or throughput times as the main outcome measure. This is perhaps unsurprising since waiting time has been shown to be a key determinant of patient satisfaction and has been strongly prioritised through the 4-hour targets.[2] Some studies[13 19 24 29] did not provide enough information on how input parameters were selected and synthesised. A handful of studies used expert opinion to populate some of the model inputs, but none explicitly justified the reason for using expert opinion. It is important to have transparent criteria for using expert opinion since it can over or under estimate the model inputs. There are several methods for eliciting expert opinion as discussed by Grigore et al.[42]

Most models were intended to capture specific aspects of the emergency care process, but some authors have argued that understanding of patient flow requires study of the entire care process.[43] Conversely, others argue that it is sufficient to focus on the specific needs of the care process rather than modelling a large and complicated care process.[25] Most of the studies (n=18; 86%) used specific hospital models of ED patient flow. Interestingly, there seemed to be no standard hospital model of patient flow of emergency care process. One generic model was developed by the Department of Health in 2007 for use across all EDs.[12] This generic approach allows hospitals to benefit from simulation methodology with minimal costs and technical expertise, but there are challenges of using a generic national model for specific local use due to the local context of each NHS hospital including differences in physical space, the demographics of local patient populations, and so on.

Just over half of the studies in our review reported some involvement of stakeholders in the simulation study. Involving stakeholders is important since it helps to understand the problem better,[8 44] assess the simulation outputs fully,[45] and translate simulation outputs into policy.[15] Very few studies reported clear summaries of whether the changes considered were supported by the simulation and of whether the changes supported were implemented. Some studies drew attention to a number of challenges particularly associated with simulation conduct and implementation. Brailsford[46] provided a helpful discussion on how to overcome the barriers such as methodological suitability, data crisis, and stakeholder issues.

Only 3 studies[12 32 34] (14%) reported on the implementation of the changes supported by the simulation outputs. This may show that the impact of computer simulation modelling within the field of UK's emergency care has been limited, though we do not know if any changes were implemented at a later date. We also do not know if any changes implemented led to any improvements in the process or outcomes of ED care. The systematic use of simulation modelling is not yet part of healthcare, whereas its use in other sectors like in manufacturing or airline industry is an integral part of the actual decision-making process.[47] Why is simulation yet to make the same

For peer review only - http://bmjopen.bmj.com/site/about/guidelines.xhtml

impact in healthcare as in other industries? Lack of stakeholders' engagement has been argued as one of the main reasons for this.[8 44 46 47] To this end, Harper et al[44] discussed the basic components of successful implementation of simulation methods in healthcare. Absence of lucid guidelines about how to use simulation methods effectively in healthcare has been argued as another reason.[48] In line with a few others,[8 44 46 47] we also argue that if simulation is to make sustained impact in healthcare, the clinicians and decision-makers must cooperate across physical and organisational boundaries and come to understand how seemingly small changes in design of processes can improve patient care.

We systematically searched eight bibliographic databases to identify studies, however our study has some limitations. Firstly, we focused on the use of computer simulation methods in the context of patient flow within EDs under the jurisdiction of UK NHS only. It would be interesting to compare the identified methods with other jurisdictions, across Europe, in the USA and Australasia, but this was beyond the scope of this study. Besides, comparing studies from different jurisdictions and arriving some consensus would be challenging since healthcare delivery is different in the UK. Secondly, we did not attempt to assess the quality of the identified simulation models since we are not aware of any guidelines to carry out this type of quality assessment. Thirdly, we neither verified whether any of the hospitals implemented the findings found from simulation experiments, nor do we know if any changes implemented led to any improvements. Finally, anecdotal evidence suggests that NHS hospitals have used simulation modelling (and other methods) to improve patient flow through the ED. However, our review will not capture all of this work as it is not all reported in academic publications.

CONCLUSIONS

We found that computer simulation can provide a means to pre-test the likely impact of changes to the delivery of emergency care before implementation in a safe and efficient manner. In particular, it is used to identify the key relationships and bottlenecks in the process of ED care, test 'what-if' scenarios for service redesign, determine levels of uncertainty, provide visualisations, and forecast future performance. However, the evidence base is small and poorly developed, with many methodological and practical issues, including lack of awareness regarding system complexity, lack of good quality data, lack of persistent engagement of stakeholders in the modelling process, lack of in-house analytical skills, and lack of an implementation plan. Furthermore, the level of detail of reporting of the computer simulation methods differed in the style and quality of reporting; and in some instances, key aspects of the assumptions underpinning the analyses were not always reported explicitly and transparently.

For peer review only - http://bmjopen.bmj.com/site/about/guidelines.xhtml

Future studies should justify the choice of simulation modelling method explicitly, avoid making selective use of the available data, engage stakeholders in the modelling process and keep them on board continually, be transparent in the reporting of simulation inputs and outputs, and report on the implementation of changes supported by the findings of simulation experiments. We recommend the adoption of reporting guidelines[41] by academic journals and conference proceedings, and more persistent exploitation of innovative models of engagement and knowledge mobilisation between academics and healthcare professionals such as the Researchers in Residence.[49] Further research is necessary to assess the quality of computer simulation models of ED patient flow across different countries and to establish the extent to which the simulation outputs have been translated into policy.

For peer review only

ACKNOWLEDGEMENTS

All the authors apart from CV are supported by the National Institute for Health Research (NIHR) Collaboration for Leadership in Applied Health Research and Care West (CLAHRC West) at University Hospitals Bristol National Health Service (NHS) Foundation Trust. The views expressed are those of the authors and not necessarily those of the NHS, the NIHR, or the Department of Health.

CONTRIBUTORS

SM and JB independently screened and appraised the relevant studies, and extracted the data from the included studies. CV, SM, JS, KN, JB, and AR designed the search strategy. AR, an information specialist, conducted the electronic search. SM drafted the manuscript in conjunction with CV. JD, WH, and CV revised the draft critically for intellectual content, while other authors commented on the draft. All authors read and approved the submitted manuscript.

FUNDING

NIHR

COMPETING INTERESTS

None

For peer review only

Table 1: Detail of the included studies

Study Name (Year)	Publication Type (Name)	Hospital Name	Simulation Purpose	ED Detail	Patient Flow Description
Anagnostou et al[19] (2013)	Conference proceedings (Winter Simulation Conference)	Unknown several hospitals in Greater London	Proof of concept	No detail	Textual; Activity list
Au-Yeung et al[20] (2006)	Conference proceedings (Modelling and Simulation)	Unknown hospital in North London	Service redesign	More detail	Flow chart; Textual
Baboolal et al[21] (2012)	Journal article (Emergency Medicine Journal)	University hospital of Wales	Service redesign	More detail	Textual
Bowers et al[22] (2009)	Journal article (Journal of Simulation)	Unknown hospital in Fife, Scotland	Service redesign	Some detail	Flow chart
Brailsford et al[10] (2004)	Journal article (Journal of Operational Research Society)	Nottingham city hospital & QMC in Nottingham	Service redesign	More detail	Textual
Coats et al[23] (2001)	Journal article (Emergency Medicine Journal)	Royal London hospital in Whitechapel, London	Service redesign	No detail	Flow chart
Codrington-Virtue et al[24] (2006)	Conference proceedings (Computer-Based Medical Systems)	Unknown hospital	Understand capacity	More detail	Textual
Codrington-Virtue et al[25] (2011)	Conference proceedings (Winter Simulation Conference)	Unknown hospital	Proof of concept	Some detail	Flow chart; Textual
Coughlan et al[26] (2011)	Journal article (Emergency Medicine Journal)	Unknown district general hospital in West London	Service redesign	Some detail	Flow chart; Textual
Davies et al[27] (2007)	Conference proceedings (Winter Simulation Conference)	Unknown hospital	Service redesign	More detail	Flow chart; Textual
Eatock et al[11] (2011)	Journal article (Journal of Health Org. and Management)	Hillingdon hospital in West London	Service redesign	More detail	Flow chart; Textual
Fletcher et al[12] (2007)	Journal article (Journal of Operational Research Society)	Unknown hospitals (n=10)	Service redesign	No detail	Flow chart; Textual
Gunal et al[28] (2006)	Conference proceedings (Winter Simulation Conference)	Unknown hospital	Service redesign	Some detail	Textual; Activity list
Gunal et al[13] (2009)	Journal article (Emergency Medicine Journal)	Unknown hospital	Understand behaviour	No detail	Flow chart; Textual
Hay et al[29] (2006)	Conference proceedings (Winter Simulation Conference)	Unknown hospitals (n=4)	Understand behaviour	No detail	Flow chart; Textual
Komashie et al[30] (2005)	Conference proceedings (Winter Simulation Conference)	Unknown hospital in London	Service redesign	More detail	Flow chart
Lane et al[31] (2000)	Journal article (Journal of Operational Research Society)	Unknown teaching hospital in London	Service redesign; Forecasting	More detail	Flow chart; Textual
Lattimer et al[14] (2004)	Journal article (Emergency Medicine Journal)	Nottingham city hospital & QMC in Nottingham	Service Redesign; Forecasting	Some detail	Flow chart
Maull et al[32] (2009)	Journal article (The Service Industries Journal)	Unknown hospital in South West of England	Service redesign; Forecasting	No detail	Flow chart
Meng et al[33] (2008)	Conference proceedings (Winter Simulation Conference)	Unknown hospital	Service redesign	More detail	Flow chart; Textual
Mould et al[34] (2013)	Journal article (Health Systems)	Unknown hospital in Fife, Scotland	Service redesign	No detail	Flow chart

QMC = Queen's Medical Centre

For peer review only - http://bmjopen.bmj.com/site/about/guidelines.xhtml

Table 2: Summary of simulation methods

Study Name	Simulation Type	Rationale for Simulation Type	Model Type	Simulation Duration	Warmup Period	Simulation Run	Simulation Software
Anagnostou et al[19]	DES	Yes	Specific	Not reported	Not reported	Not reported	RePastSimphony
Au-Yeung et al[20]	DES[a]	Yes	Specific	Not reported	Not reported	10	Written in Java
Baboolal et al[21]	DES	Yes	Specific	Not reported	Not reported	Not reported	Simul8
Bowers et al[22]	DES	Yes	Specific	Not reported	Not reported	Not reported	Not reported
Brailsford et al[10]	DES[b]	Yes	Specific	Not reported	Not reported	Not reported	Simul8
Coats et al[23]	DES	No	Specific	Not reported	Not reported	Not reported	Simul8
Codrington-Virtue et al[24]	DES	Yes	Specific	52 weeks	Not reported	Not reported	Simul8
Codrington-Virtue et al[25]	DES	Yes	Specific	24 hours	24 hours	50	Simul8
Coughlan et al[26]	DES	Yes	Specific	3 weeks	Not reported	Not reported	Simul8
Davies et al[27]	DES	Yes	Specific	Not reported	Not reported	Not reported	Simul8
Eatock et al[11]	DES	Yes	Specific	3 weeks	Not reported	20	Simul8
Fletcher et al[12]	DES	Yes	Generic	Not reported	Not reported	Not reported	Simul8
Gunal et al[28]	DES	Yes	Generic	52 weeks	0	50	Micro Saint Sharp
Gunal et al[13]	DES	Yes	Generic	Not reported	Not reported	Not reported	Micro Saint Sharp
Hay et al[29]	DES	Yes	Specific	Not reported	Not reported	Not reported	Arena
Komashie et al[30]	DES	Yes	Specific	Not reported	Not reported	Not reported	Arena
Lane et al[31]	SD	Yes	Specific	24 hours	Not reported	6	iThink
Lattimer et al[14]	SD	Yes	Specific	52 weeks	Not reported	Not reported	Stella
Maull et al[32]	DES	Yes	Specific	Not reported	Not reported	Not reported	Not reported
Meng et al[33]	DES	Yes	Specific	Not reported	Not reported	Not reported	MedModel
Mould et al[34]	DES	Yes	Specific	3 months	24 hours	Not reported	Simul8

DES = Discrete Event Simulation
SD = System Dynamics
[a] the authors used a Markovian queueing network, but computed the moments and densities of patient treatment time through a DES
[b] the authors used an SD model as part of a bigger picture, but modelled the A&E through a DES

For peer review only - http://bmjopen.bmj.com/site/about/guidelines.xhtml

Table 3: Detail of simulation inputs and outputs

Study Name	Source of Arrival Rates	Source of Activity Duration	Source of Activity Progression	Source of Resources Use	Changes Considered	Outcomes Considered	Validation	Stakeholder Input
Anagnostou et al[19]	Not described	Not described	Not described	Not described	None	Waiting times	None	None
Au-Yeung et al[20]	Primary	Primary; Expert opinion	Primary; Expert opinion	Primary	ED patient flow	Waiting times	Data-led	Model specification
Baboolal et al[21]	Primary	Primary; Expert opinion	Primary	Primary	Resources	Waiting times*; Resources	Face<br>Dark world model	Model specification
Bowers et al[22]	Primary	Primary	Primary	Not described	None	Resources*	Data-led<br>Face	Model specification
Brailsford et al[10]	Primary	Secondary	Not described	Primary	ED patient flow; Arrival rates	Waiting times	Data-led<br>Face	Study question; Model specification
Coats et al[23]	Primary	Primary	Not described	Not described	Shift patterns	Waiting times*	Data-led	None
Codrington-Virtue et al[24]	Primary	Not described	Not described	Not described	None	Resources	None	None
Codrington-Virtue et al[25]	Primary	Primary; Expert opinion	Primary	Primary; Expert opinion	None	Waiting times	Data-led	None
Coughlan et al[26]	Primary	Not described	Primary	Primary	Resources	Waiting times*; Resources	Data-led	None
Davies et al[27]	Primary	Primary	Primary	Primary	ED patient flow	Waiting times*	None	None
Eatock et al[11]	Primary	Primary; Expert opinion	Primary; Expert opinion	Primary	None	Waiting times*	Data-led	None
Fletcher et al[12]	Primary	Secondary; Expert opinion	Primary	Primary	ED patient flow; Resources; Demand	Waiting times*; Resources	Data-led<br>Face	Study question; Model specification
Gunal et al[28]	Primary	Primary	Primary	Not described	ED patient flow; Resources	Waiting times*	Data-led	Model specification
Gunal et al[13]	Primary	Not described	Not described	Not described	None	Waiting times*	None	None
Hay et al[29]	Primary	Not described	Not described	Not described	None	Waiting times*	Data-led	None
Komashie et al[30]	Primary	Primary; Expert opinion	Primary; Expert opinion	Primary; Expert opinion	ED structure; Resources	Waiting times; Resources	Data-led<br>Face	Study question; Model specification
Lane et al[31]	Primary	Primary; Expert opinion	Expert opinion	Primary	Resources; Demand	Waiting times; Resources; Elective cancellations	Data-led<br>Face	Study question; Model specification
Lattimer et al[14]	Primary	Primary	Primary	Primary	ED Structure; Admission rates	Bed occupancy	Data-led<br>Face	Study question; Model specification
Maull et al[32]	Primary	Primary; Expert opinion	Primary	Not described	ED patient flow	Waiting times*	Data-led	Result implementation
Meng et al[33]	Primary	Primary	Not described	Not described	ED structure; Resources	Waiting times; Resources	Data-led<br>Face	Study question; Model specification
Mould et al[34]	Primary	Primary	Primary	Not described	Resources	Waiting times*; Resources	Data-led	Study question; Model specification; Result implementation

* these studies used 4-hour target breach as part of their waiting time considerations

For peer review only - http://bmjopen.bmj.com/site/about/guidelines.xhtml

Table 4: Summary of simulation results

Study Name	Conclusions	Conclusions Detail	Reported the Changes Implemented?	Result Implementation	Barriers
Anagnostou et al[19]	None	None	No	N/A	None
Au-Yeung et al[20]	Supported the changes considered	Prioritisation of treatment for patient with minor problems over major problems could lead to improved outcome	No	N/A	Simplified assumptions
Baboolal et al[21]	Supported the changes considered	A change in staffing levels could lead to substantial cost savings and reduce the 4-hour breaches	No	N/A	None
Bowers et al[22]	None	None	No	N/A	Model runtime; High expectancy
Brailsford et al[10]	Opposed the changes considered	Streaming of patients by triage category was not an efficient use of clinical resources	No	N/A	None
Coats et al[23]	Supported the changes considered	Shift pattern that best matches patient arrivals would give shorter waiting times	No	N/A	Simplified model structure and assumptions; Poor data quality
Codrington-Virtue et al[24]	None	None	No	N/A	None
Codrington-Virtue et al[25]	None	None	No	N/A	None
Coughlan et al[26]	Proposed differential changes	Adding an emergency nurse practitioner would not reduce the waiting times. Resource reallocation would improve throughput times	No	N/A	Generalisability
Davies et al[27]	Supported the changes considered	The separation of see and treat would be beneficial	No	N/A	Poor data quality
Eatock et al[11]	None	None	No	N/A	System complexity; Model runtime
Fletcher et al[12]	Proposed differential changes	Deflecting demand away from A&E would lead to improvement around waiting for beds, specialists, and assessment processes	Yes	Unknown as other interventions were introduced in parallel	Poor data quality; Poor stakeholder engagement
Gunal et al[28]	Proposed differential changes	More senior doctors, less x-ray requisitions, and more cubicles would reduce waiting times	No	N/A	Modelling multitasking behaviour of staff
Gunal et al[13]	None	None	No	N/A	Explaining the causes of change in performance
Hay et al[29]	None	None	No	N/A	System complexity
Komashie et al[30]	Proposed differential changes	Adding a nurse or doctor to minors would reduce the waiting times by 28%. Increasing the cubicles/beds would make smaller change	No	N/A	None
Lane et al[31]	Proposed differential changes	Changing bed numbers led to no noticeable change in waiting times but a substantial difference to elective cancelations	No	N/A	Short-timescale; Simplified assumptions
Lattimer et al[14]	Proposed differential changes	System would not be able to cope with increasing demand from scenario 1[a], but scenario 2[b], 3[c], and 4[d] could improve this	No	N/A	Simplified model structure; System complexity; Generalisability
Maull et al[32]	Supported the changes considered	See and treat reduced the 4-hour breaches from 13.2% to 3.4%	Yes	Marked reduction in no. of breaches from 13.2% to 1.4%. No. of patients waiting less than 1-hour increased from 12% to 23%. No. of patients with major problems waiting between 3 and 4 hours increased	Poor data availability and quality; System complexity
Meng et al[33]	Proposed differential changes	Reduced times to see a consultant would reduce the waiting times. Access to 24-hour x-ray would reduce the waiting times too	No	N/A	Simplified assumptions
Mould et al[34]	Supported the changes considered	A new staff roster would reduce the waiting times	Yes	Mean time for minor problems dropped from 100 to 94 mins, for major problems it dropped from 200 to 195 mins. Mean time for minor problems fell by 16 mins	Poor data quality; Limited analytical skills; Impact of simulation

For peer review only - http://bmjopen.bmj.com/site/about/guidelines.xhtml

				after adjusting other factors	

[a] 5-year model run assuming 4% year on year growth in emergency admissions and 3% year on year growth in GP referral for planned admissions

[b] impact of increase in demand for front door services

[c] reducing emergency admissions of patients with respiratory or coronary problems, ill-defined conditions, and over 65 years

[d] effects of earlier discharge of patients admitted as emergencies and subsequently discharged to nursing or residential homes

For peer review only

For peer review only - http://bmjopen.bmj.com/site/about/guidelines.xhtml

REFERENCES

1. Banerjee A, Mbamalu D, Hinchley G. The impact of process re-engineering on patient throughput in emergency departments in the UK. *Int J Emerg Med* 2008;1(3):189-92. doi: 10.1007/s12245-008-0055-x
2. Hemaya SA, Locker TE. How accurate are predicted waiting times, determined upon a patient's arrival in the Emergency Department? *Emergency Medicine Journal* 2012;29(4):316-18.
3. Trzeciak S, Rivers EP. Emergency department overcrowding in the United States: an emerging threat to patient safety and public health. *Emerg Med J* 2003;20(5):402-5.
4. Miro O, Antonio MT, Jimenez S, et al. Decreased health care quality associated with emergency department overcrowding. *Eur J Emerg Med* 1999;6(2):105-7.
5. Trout A, Magnusson AR, Hedges JR. Patient satisfaction investigations and the emergency department: what does the literature say? *Acad Emerg Med* 2000;7(6):695-709.
6. Fatovich DM, Hirsch RL. Entry overload, emergency department overcrowding, and ambulance bypass. *Emerg Med J* 2003;20(5):406-9.
7. Blunt I. Focus on: A&E attendances. Why are patients waiting longer? Quality Watch, The Health Foundation and Nuffield Trust. 2014 [31 August 2016]. Available from: http://www.nuffieldtrust.org.uk/sites/files/nuffield/publication/140724_focus_on_ae_attendances.pdf.
8. Carrus B, Corbett S, Khandelwal D. A hospital-wide strategy for fixing emergency-department overcrowding. *McKinsey Quarterly* 2010:1-12.
9. de Koning H, Verver JP, van den Heuvel J, et al. Lean six sigma in healthcare. *J Healthc Qual* 2006;28(2):4-11.
10. Brailsford SC, Lattimer VA, Tarnaras P, et al. Emergency and on-demand health care: modelling a large complex system. *J Oper Res Soc* 2004;55(1):34-42. doi: 10.1057/palgrave.jors.2601667
11. Eatock J, Clarke M, Picton C, et al. Meeting the four-hour deadline in an A&E department. *Journal of health organization and management* 2011;25(6):606-24.
12. Fletcher A, Halsall D, Huxham S, et al. The DH Accident and Emergency Department model: a national generic model used locally. *J Oper Res Soc* 2007;58(12):1554-62. doi: DOI 10.1057/palgrave.jors.2602344
13. Gunal MM, Pidd M. Understanding target-driven action in emergency department performance using simulation. *Emergency Medicine Journal* 2009;26(10):724-27. doi: 10.1136/emj.2008.066969
14. Lattimer V, Brailsford S, Turnbull J, et al. Reviewing emergency care systems I: insights from system dynamics modelling. *Emergency Medicine Journal* 2004;21(6):685-91. doi: 10.1136/emj.2002.003673
15. Sobolev B, Kuramoto L. Policy analysis using patient flow simulations: Conceptual framework and study design. *Clin Invest Med* 2005;28(6):359-63.
16. Pitt M, Monks T, Crowe S, et al. Systems modelling and simulation in health service design, delivery and decision making. *BMJ Qual Saf* 2016;25(1):38-45. doi: 10.1136/bmjqs-2015-004430
17. Davies R, Roderick P, Raftery J. The evaluation of disease prevention and treatment using simulation models. *Eur J Oper Res* 2003;150(1):53-66. doi: 10.1016/S0377-2217(02)00783-X
18. Brailsford S, Hilton N. A comparison of discrete event simulation and system dynamics for modelling health care systems. 2001
19. Anagnostou A, Nouman A, Taylor SJE. Distributed Hybrid Agent-Based Discrete Event Emergency Medical Services Simulation. *Wint Simul C Proc* 2013:1625-36.
20. Au-Yeung SWM, Harrison PG, Knottenbelt WJ. A queueing network model of patient flow in an accident and emergency department. *Modelling and Simulation 2006* 2006:60-67.
21. Baboolal K, Griffiths JD, Knight VA, et al. How efficient can an emergency unit be? A perfect world model. *Emergency Medicine Journal* 2012;29(12):972-77.
22. Bowers J, Ghattas M, Mould G. Success and failure in the simulation of an accident and emergency department. *Journal of Simulation* 2009;3(3):171-78.

For peer review only

23. Coats TJ, Michalis S. Mathematical modelling of patient flow through an accident and emergency department. *Emergency Medicine Journal* 2001;18(3):190-92. doi: DOI 10.1136/emj.18.3.190
24. Codrington-Virtue A, Chaussalet T, Millard P, et al. A system for patient management based discrete-event simulation and hierarchical clustering. *Comp Med Sy* 2006:800-04. doi: Doi 10.1109/Cbms.2006.31
25. Codrington-Virtue A, Kelly J, Chaussalet T. Using Simplified Discrete-Event Simulation Models for Health Care Applications. *Proceedings of the 2011 Winter Simulation Conference (WSC)* 2011:1154-65.
26. Coughlan J, Eatock J, Patel N. Simulating the use of re-prioritisation as a wait-reduction strategy in an emergency department. *Emergency Medicine Journal* 2011;28(12):1013-18. doi: 10.1136/emj.2010.100255
27. “See and Treat” or “See” and “Treat” in an emergency department. 2007 Winter Simulation Conference; 2007. IEEE.
28. Gunal MM, Pidd M. Understanding accident and emergency department performance using simulation. *Proceedings of the 2006 Winter Simulation Conference, Vols 1-5* 2006:446-52. doi: Doi 10.1109/Wsc.2006.323114
29. Modeling emergency care in hospitals: a paradox-the patient should not drive the process. Proceedings of the 2006 Winter Simulation Conference; 2006. IEEE.
30. Modeling emergency departments using discrete event simulation techniques. Proceedings of the 37th conference on Winter simulation; 2005. Winter Simulation Conference.
31. Lane DC, Monefeldt C, Rosenhead JV. Looking in the wrong place for healthcare improvements: A system dynamics study of an accident and emergency department. *J Oper Res Soc* 2000;51(5):518-31. doi: Doi 10.2307/254183
32. Maull RS, Smart P, Harris A, et al. An evaluation of ‘fast track’in A&E: a discrete event simulation approach. *The Service Industries Journal* 2009;29(7):923-41.
33. Meng LY, Spedding T. Modelling Patient Arrivals When Simulating an Accident and Emergency Unit. *2008 Winter Simulation Conference, Vols 1-5* 2008:1509-15.
34. Mould G, Bowers J, Dewar C, et al. Assessing the impact of systems modeling in the redesign of an Emergency Department. *Health Systems* 2013;2(1):3-10.
35. Interconnected DES models of emergency, outpatient, and inpatient departments of a hospital. 2007 Winter simulation conference; 2007. IEEE.
36. Günal MM, Pidd M. DGHPSIM:: Generic simulation of hospital performance. *ACM Transactions on Modeling and Computer Simulation (TOMACS)* 2011;21(4):23.
37. Lane DC, Monefeldt C, Husemann E. Client involvement in simulation model building: hints and insights from a case study in a London hospital. *Health Care Manag Sci* 2003;6(2):105-16.
38. Mould G, Bowers J. Roles of pathway-based models and their contribution to the redesign of health-care systems. *International Journal of Care Pathways* 2011;15(3):90-97.
39. Balci O. Validation, verification, and testing techniques throughout the life cycle of a simulation study. *Annals of operations research* 1994;53(1):121-73.
40. Robinson S. Simulation: the practice of model development and use. *Hoboken, NJ: John Wiley & Sons* 2004
41. Karnon J, Stahl J, Brennan A, et al. Modeling using discrete event simulation: a report of the ISPOR-SMDM Modeling Good Research Practices Task Force--4. *Value Health* 2012;15(6):821-7. doi: 10.1016/j.jval.2012.04.013
42. Grigore B, Peters J, Hyde C, et al. A comparison of two methods for expert elicitation in health technology assessments. *BMC Med Res Methodol* 2016;16:85. doi: 10.1186/s12874-016-0186-3
43. Haraden C, Resar R. Patient flow in hospitals: understanding and controlling it better. *Front Health Serv Manage* 2004;20(4):3-15.
44. Harper PR, Pitt M. On the challenges of healthcare modelling and a proposed project life cycle for successful implementation. *J Oper Res Soc* 2004;55(6):657-61.
45. Eldabi T, Irani Z, Paul RJ. A proposed approach for modelling health-care systems for understanding. *J Manag Med* 2002;16(2-3):170-87.

For peer review only - http://bmjopen.bmj.com/site/about/guidelines.xhtml

46. Brailsford S. Overcoming the barriers to implementation of operations research simulation models in healthcare. *Clin Invest Med* 2005;28(6):312-5.
47. Eldabi T, Paul R, Young T. Simulation modelling in healthcare: reviewing legacies and investigating futures. *J Oper Res Soc* 2007;58(2):262-70.
48. Jun GT, Ward J, Morris Z, et al. Health care process modelling: which method when? *International Journal for Quality in Health Care* 2009;21(3):214-24.
49. Marshall M, Pagel C, French C, et al. Moving improvement research closer to practice: the Researcher-in-Residence model. *BMJ Qual Saf* 2014;23(10):801-5. doi: 10.1136/bmjqs-2013-002779

For peer review only

For peer review only - http://bmjopen.bmj.com/site/about/guidelines.xhtml

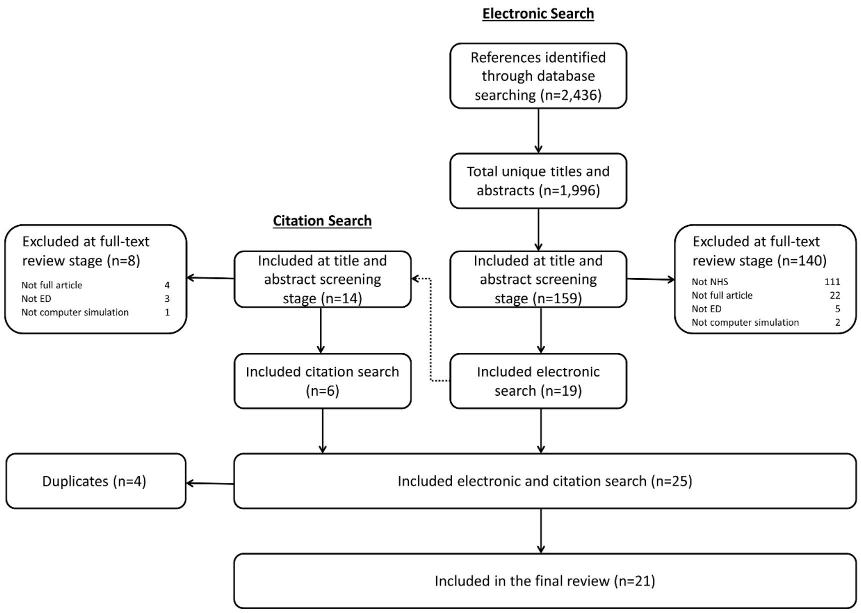

Figure 1 Flowchart of the study identification and inclusion process.

400x400mm (300 x 300 DPI)

APPENDIX 1

MEDLINE search strategy

----

1 exp Computer Simulation/ (175146)

2 models, statistical/ (75054)

3 ((computer* or distributed or hybrid) adj6 (model* or simulation*)).mp. (184347)

4 ((flow or simulation) adj6 model$).mp. (41697)

5 (system adj3 model$).mp. (50666)

6 (discrete adj2 event).mp. (783)

7 (monte adj2 carlo).mp. (40800)

8 markov.mp. (17983)

9 (statechart$ or stochastic or system dynamics).mp. (32076)

10 (agent adj2 based).mp. (3106)

11 or/1-10 (391259)

12 workflows/ or crowding/ or exp waiting lists/ (13340)

13 time to treatment/ or triage/ or patient admission/ or length of stay/ (92230)

14 exp critical pathways/ or exp "Process Assessment (Health care)"/ (8528)

15 exp patient care planning/ or exp patient care/ or exp "continuity of patient care"/ (679418)

16 ((clinical or critical or patient*) adj2 (path or paths or pathways)).tw. (5722)

17 (patient$ adj3 (flow or flows or flowing or throughput)).tw. (11371)

18 (care adj3 (step or steps or stage$)).tw. (2424)

19 (process adj2 care).tw. (4165)

20 (workflow* or crowding* or waiting list* or waiting time*).tw. (31590)

21 or/12-20 (741748)

22 emergency medical services/ or emergency medical service communication systems/ or emergency service, hospital/ or exp ambulances/ (86523)

23 ((emergency or emergencies or unplanned or unscheduled) adj3 (care or admission$ or department$ or ward or wards or service$ or unit or units or room or rooms)).tw. (95179)

24 (A&E or "accident and emergency").tw. (18002)

25 (minor injur* adj2 (unit* or servic* or ward* or hospital* or department*)).ti,ab. (137)

26 22 or 23 or 24 or 25 (154509)

27 11 and 21 and 26 (437)

For peer review only

PRISMA 2009 Checklist

Section/topic	#	Checklist item	Reported on page #
**TITLE**			
Title	1	Identify the report as a systematic review, meta-analysis, or both.	1
**ABSTRACT**			
Structured summary	2	Provide a structured summary including, as applicable: background; objectives; data sources; study eligibility criteria, participants, and interventions; study appraisal and synthesis methods; results; limitations; conclusions and implications of key findings; systematic review registration number.	2
**INTRODUCTION**			
Rationale	3	Describe the rationale for the review in the context of what is already known.	5
Objectives	4	Provide an explicit statement of questions being addressed with reference to participants, interventions, comparisons, outcomes, and study design (PICOS).	5-6
**METHODS**			
Protocol and registration	5	Indicate if a review protocol exists, if and where it can be accessed (e.g., Web address), and, if available, provide registration information including registration number.	6
Eligibility criteria	6	Specify study characteristics (e.g., PICOS, length of follow-up) and report characteristics (e.g., years considered, language, publication status) used as criteria for eligibility, giving rationale.	6
Information sources	7	Describe all information sources (e.g., databases with dates of coverage, contact with study authors to identify additional studies) in the search and date last searched.	6
Search	8	Present full electronic search strategy for at least one database, including any limits used, such that it could be repeated.	Appendix 1
Study selection	9	State the process for selecting studies (i.e., screening, eligibility, included in systematic review, and, if applicable, included in the meta-analysis).	6-7
Data collection process	10	Describe method of data extraction from reports (e.g., piloted forms, independently, in duplicate) and any processes for obtaining and confirming data from investigators.	7
Data items	11	List and define all variables for which data were sought (e.g., PICOS, funding sources) and any assumptions and simplifications made.	7
Risk of bias in individual studies	12	Describe methods used for assessing risk of bias of individual studies (including specification of whether this was done at the study or outcome level), and how this information is to be used in any data synthesis.	N/A
Summary measures	13	State the principal summary measures (e.g., risk ratio, difference in means).	N/A
Synthesis of results	14	Describe the methods of handling data and combining results of studies, if done, including measures of consistency (e.g., I^2) for each meta-analysis.	N/A

For peer review only - http://bmjopen.bmj.com/site/about/guidelines.xhtml

PRISMA 2009 Checklist

Section/topic	#	Checklist item	Reported on page #
Risk of bias across studies	15	Specify any assessment of risk of bias that may affect the cumulative evidence (e.g., publication bias, selective reporting within studies).	N/A
Additional analyses	16	Describe methods of additional analyses (e.g., sensitivity or subgroup analyses, meta-regression), if done, indicating which were pre-specified.	N/A
**RESULTS**			
Study selection	17	Give numbers of studies screened, assessed for eligibility, and included in the review, with reasons for exclusions at each stage, ideally with a flow diagram.	7-8<br>Fig. 1
Study characteristics	18	For each study, present characteristics for which data were extracted (e.g., study size, PICOS, follow-up period) and provide the citations.	8
Risk of bias within studies	19	Present data on risk of bias of each study and, if available, any outcome level assessment (see item 12).	N/A
Results of individual studies	20	For all outcomes considered (benefits or harms), present, for each study: (a) simple summary data for each intervention group (b) effect estimates and confidence intervals, ideally with a forest plot.	Tables 1-4
Synthesis of results	21	Present results of each meta-analysis done, including confidence intervals and measures of consistency.	N/A
Risk of bias across studies	22	Present results of any assessment of risk of bias across studies (see Item 15).	N/A
Additional analysis	23	Give results of additional analyses, if done (e.g., sensitivity or subgroup analyses, meta-regression [see Item 16]).	N/A
**DISCUSSION**			
Summary of evidence	24	Summarize the main findings including the strength of evidence for each main outcome; consider their relevance to key groups (e.g., healthcare providers, users, and policy makers).	10-11
Limitations	25	Discuss limitations at study and outcome level (e.g., risk of bias), and at review-level (e.g., incomplete retrieval of identified research, reporting bias).	12
Conclusions	26	Provide a general interpretation of the results in the context of other evidence, and implications for future research.	12-13
**FUNDING**			
Funding	27	Describe sources of funding for the systematic review and other support (e.g., supply of data); role of funders for the systematic review.	14

From: Moher D, Liberati A, Tetzlaff J, Altman DG, The PRISMA Group (2009). Preferred Reporting Items for Systematic Reviews and Meta-Analyses: The PRISMA Statement. PLoS Med 6(7): e1000097. doi:10.1371/journal.pmed1000097

For more information, visit: www.prisma-statement.org.

For peer review only - http://bmjopen.bmj.com/site/about/guidelines.xhtml

BMJ Open

A systematic review of the use of computer simulation modelling of patient flow within UK emergency departments

Journal:	*BMJ Open*
Manuscript ID	bmjopen-2016-015007.R1
Article Type:	Research
Date Submitted by the Author:	07-Feb-2017

Complete List of Authors:	Mohiuddin, Syed; University Hospitals Bristol NHS Foundation Trust, NIHR CLAHRC West; University of Bristol, School of Social and Community Medicine<br>Busby, John; Queen's University Belfast, School of Medicine<br>Savovic, Jelena; University Hospitals Bristol NHS Foundation Trust, NIHR CLAHRC West; University of Bristol, School of Social and Community Medicine<br>Richards, Alison; University Hospitals Bristol NHS Foundation Trust, NIHR CLAHRC West; University of Bristol, School of Social and Community Medicine<br>Northstone, Kate; University Hospitals Bristol NHS Foundation Trust, NIHR CLAHRC West; University of Bristol, School of Social and Community Medicine<br>Hollingworth, William; University Hospitals Bristol NHS Foundation Trust, NIHR CLAHRC West; University of Bristol, School of Social and Community Medicine<br>Donovan, Jenny; University Hospitals Bristol NHS Foundation Trust, NIHR CLAHRC West; University of Bristol, School of Social and Community Medicine<br>Vasilakis, C; University of Bath, School of Management

**Primary Subject Heading**:	Health services research
Secondary Subject Heading:	Emergency medicine, Communication, Health policy, Medical management, Research methods
Keywords:	Systematic review, Emergency care, Overcrowding, Computer simulation, Patient flow, Waiting times

SCHOLARONE™ Manuscripts

A systematic review of the use of computer simulation modelling of patient flow within UK emergency departments

Syed Mohiuddin,[1,2*] John Busby,[3] Jelena Savović,[1,2] Alison Richards,[1,2] Kate Northstone,[1,2] William Hollingworth,[1,2] Jenny L. Donovan,[1,2] Christos Vasilakis[4]

[1]NIHR CLAHRC West, University Hospitals Bristol NHS Foundation Trust, Bristol, UK

[2]School of Social and Community Medicine, University of Bristol, Bristol, UK

[3]School of Medicine, Queen's University Belfast, Belfast, UK

[4]School of Management, University of Bath, Bath, UK

[*]corresponding author:

Address: Whitefriars (9th Floor), Bristol BS1 2NT, UK

E-mail: syed.mohiuddin@bristol.ac.uk

Telephone: +44 (0)117 342 1267

Keywords:

Systematic review; Emergency care; Overcrowding; Computer simulation; Patient flow; Waiting times

Word count:

3,996

For peer review only

ABSTRACT

Objectives: Overcrowding in the emergency department (ED) is common in the UK as in other countries worldwide. Computer simulation is one approach used for understanding the causes of ED overcrowding and assessing the likely impact of changes to the delivery of emergency care. However, little is known about the usefulness of computer simulation for analysis of ED patient flow. We undertook a systematic review to investigate the different computer simulation methods and their contribution for analysis of patient flow within EDs in the UK.

Methods: We searched eight bibliographic databases (MEDLINE; EMBASE; COCHRANE; WEB OF SCIENCE; CINAHL; INSPEC; MATHSCINET; and ACM DIGITAL LIBRARY) from date of inception until 31 March 2016. Studies were included if they used a computer simulation method to capture patient progression within the ED of an established UK NHS hospital. Studies were summarised in terms of simulation method, key assumptions, input and output data, conclusions drawn, and implementation of results.

Results: Twenty one studies met the inclusion criteria. Of these, 19 used discrete event simulation and 2 used system dynamics models. The purpose of many of these studies (n=16; 76%) centred on service redesign. Seven studies (33%) provided no details about the ED being investigated. Most studies (n=18; 86%) used specific hospital models of ED patient flow. Overall, the reporting of underlying modelling assumptions was poor. Nineteen studies (90%) considered patient waiting or throughput times as the key outcome measure. Twelve studies (57%) reported some involvement of stakeholders in the simulation study. However, only 3 studies (14%) reported on the implementation of changes supported by the simulation.

Conclusions: We found that computer simulation can provide a means to pre-test changes to ED care delivery before implementation in a safe and efficient manner. However, there are some methodological, data, stakeholder, implementation, and reporting issues, which must be addressed by future studies.

For peer review only - http://bmjopen.bmj.com/site/about/guidelines.xhtml

Strengths and limitations of this study

- We systematically reviewed the peer-reviewed literature to investigate the contribution of various computer simulation methods for analysis of patient flow within EDs in the UK.
- We searched eight bibliographic databases to identify the relevant studies. Further to the electronic search, we conducted backward and forward citation searches of all included studies.
- We highlighted a number of methodological, data, stakeholder, implementation, and reporting related issues associated with current studies.
- We included studies which were conducted under the jurisdiction of UK NHS only.
- We were not aware of any formal assessment checklist to estimate quality scores, nonetheless we assessed the key components of methodological quality of all included studies.

For peer review only

For peer review only - http://bmjopen.bmj.com/site/about/guidelines.xhtml

INTRODUCTION

An emergency department (ED), also known as accident & emergency department (A&E), provides acute care for patients who attend hospital without prior appointment. The EDs of most hospitals customarily operate 24 hours a day, 7 days a week. Nevertheless, overcrowding in EDs is an increasing problem in countries around the world, and especially so in the United Kingdom (UK).[1 2] ED overcrowding has been shown to have many adverse consequences such as increased medical errors,[3] decreased quality of care and subsequently poor patient outcomes,[4] increased workload,[1] frustration among ED staff,[4 5] ambulance diversions,[6] increased patient dissatisfaction,[5] prolonged patient waiting times,[7] and increased cost of care.[8] Furthermore, some less severely ill patients may leave without being seen by a physician, only to return later with a more complicated condition.[8]

In the UK, there is an enormous pressure from public and government to alleviate overcrowding and long waiting times experienced in ED.[1] The Department of Health set a target standard for acute hospitals in the National Health Service (NHS) that at least 95% of patients attending an A&E department must be seen, treated, admitted, or discharged in under four hours.[7] This 4-hour target standard was initially set at 98% in 2004, but later reduced to 95% in 2010. Beyond target setting, it has been argued that ED overcrowding can be improved by lean healthcare thinking with a focus on improving patient flow.[1 9]

Over recent decades, computer simulation and other modelling methods have been used to analyse ED patient flow and resource capacity planning.[10-14] In essence, a computer simulation model is a simplified representation of reality used to aid the understanding of the key relationships and dynamics in the care process, and to evaluate the likely impact of changes before implementation. Typically, a simulation model is based on the notion that each simulated individual is tracked through the care process; the population effect is then estimated from the sum of the individual effects.[15]

The precise way in which a simulation model works depends on the type of simulation method used. Generally, simulation models can be categorised as static or dynamic, as stochastic or deterministic, and as discrete-time or continuous-time.[16] A static simulation represents a process at a particular point in time, whereas a dynamic simulation represents a process as it evolves over time. A simulation model in which at least one input parameter is a random variable is said to be stochastic, whereas a simulation model having no random variables is said to be deterministic. A discrete-time model is one in which the state variables change instantaneously at discrete points in time. In contrast, a continuous-time model is one in which the state variables change continuously with respect to time. The advancement of computer technology has undoubtedly supported the utilisation of more sophisticated simulation methods for modelling healthcare processes. Today, for

For peer review only - http://bmjopen.bmj.com/site/about/guidelines.xhtml

example, computer simulation is also capable of providing an insight into the workings of a system through visual animation.

Various types of computer simulation exist, including discrete event simulation (DES), system dynamics (SD), and agent based simulation (ABS). DES is a widely used method, and can replicate the behaviour of complex healthcare systems over time. A DES model is a network of queues and activities (such as having a blood test, x-ray, and treatment). One of the major advantages of using a DES model is its flexibility to model complex scenarios at the individual level. Within a DES model, individuals move from one activity to another in sequential order at a particular point in time. Typically, the individuals enter a system and visit some of the activities (not necessarily only once) before leaving the system. The variables that govern the movement of modelled individuals (e.g. arrival rate, duration of treatment, etc.) can be random and thus readily capture the variation that is inherent in healthcare. As such, a DES model is considered particularly suitable for modelling queuing systems. This simulation method is able to incorporate life histories and complex scenarios at the individual level to influence the care pathway taken and the time between events, but specialist analytical knowledge is required typically to achieve a greater flexibility.[17]

Another widely used simulation method is system dynamics, which is used to understand the behaviour of complex healthcare systems over time through capturing aggregate (instead of individual) flows of patients. An SD model is essentially collection of stocks and flows between them. Stocks are basic stores of quantities over time, e.g. number of patients with a disease or in a particular part of a hospital department. Flows define the movement of objects between different stocks over time. Unlike DES, system dynamics does not lend itself readily to including random variables and thus input parameters are given as simple rates in the majority of studies. As such, SD is considered not the ideal method of choice for modelling a closely focussed system that involves resource-constrained queueing networks, such as an ED.[10] In a situation like this, DES should rather be the method of choice to model high level of distinct detail.[18] ABS is another method that has more recently been used in modelling the healthcare systems. As a new method in this application area, ABS is often overlooked in favour of using more established methods of DES and SD. The usefulness and practicalities of ABS in modelling patient flow is not well-understood.[19]

Increasing interest in this area is reflected in the number of computer simulation studies of ED patient flow and resource capacity planning that have been published over recent decades. However, little is known about the usefulness of different computer simulation methods for analysis of any changes to the delivery of emergency care. We, therefore, systematically investigated the peer-reviewed literature on the use of computer simulation modelling of patient flow within EDs in the UK. Our specific objectives were as follows:

For peer review only - http://bmjopen.bmj.com/site/about/guidelines.xhtml

i. to investigate the contribution that computer simulation studies make to our understanding of the problem of ED overcrowding;
ii. to identify the methodology used to conduct patient flow simulation in terms of key assumptions, systems requirements, and input and output data;
iii. to assess the usefulness of each simulation method for service redesign and evaluating the likely impact of changes related to the delivery of emergency care;
iv. to report on differences in conclusions about ED performance with different simulation modelling methods; and
v. to identify studies that explicitly aimed to meet the pre-specified needs of stakeholders.

METHODS

We conducted a systematic review of the peer-reviewed literature to identify computer simulation studies of patient flow within hospital EDs in the UK. This review complies with the PRISMA checklist (www.prisma-statement.org). We produced a review protocol (available from the corresponding author on request) and set out the process to address our specific objectives.

Search strategy

We retrieved relevant studies from the following bibliographic databases: MEDLINE; EMBASE; COCHRANE; WEB OF SCIENCE; CINAHL; INSPEC; MATHSCINET; and ACM DIGITAL LIBRARY. We used a key review paper[20] to select these databases, which were searched from the date of their inception until 31 March 2016. A search strategy was designed to explore three main domains of knowledge associated with the area of our interest: computer simulation; emergency care; and patient flow. We included a wide range of search phrases, both keywords and medical subject headings, such as 'computer simulation', 'emergency department', 'patient care', 'patient flow', 'waiting time', 'time to treatment', and 'length of stay'.

We first developed the search strategy for MEDLINE since it is known to allow a rich taxonomy of subjects and rubrics. We used a key review paper[20] to inform the MEDLINE search strategy and made further refinements using other relevant studies to improve sensitivity. Appendix 1 shows the MEDLINE search strategy and results from 1946 to end of March 2016. We adapted the MEDLINE strategy to search the other databases (available from the corresponding author on request). We also conducted backward and forward citation searches of all included studies using Google Scholar.

Inclusion criteria

For peer review only - http://bmjopen.bmj.com/site/about/guidelines.xhtml

We identified studies as being eligible for inclusion if they:

i. were published in peer-reviewed journals or conference proceedings as full papers;
ii. were conducted within the ED of an established UK NHS hospital responsible for assessing and treating civilians in need of emergency care;
iii. captured the progress of patients through at least two activities of an ED care process; and
iv. used a computer simulation method such as discrete event simulation, system dynamics, agent based simulation, hybrid simulation, Monte Carlo simulation, distributed simulation, or stochastic modelling.

We excluded editorials, letters, commentaries, conference abstracts, notes, and books. We also excluded studies that used methods such as regression analysis, likelihood ratio test, time series analysis, generalised linear model, mathematical programming, optimisation methods, queuing theory, structural equation modelling, process mapping, problem structuring method, or risk analysis without combining it with a computer simulation method.

Selection of studies for full-text review

To identify the studies suitable for full-text review, two authors (SM and JB) independently screened the titles and abstracts of all the initially retrieved studies. The individual responses from each reviewer were stored in a common database. At this stage, a study was excluded if it was clearly irrelevant based on our inclusion criteria. In cases of discrepancy, we selected the studies for full-text review by consensus.

Appraisal of studies for inclusion

An electronic questionnaire was designed to appraise the studies selected for full-text review. The questionnaire included four key questions: (i) is the study a full paper published in a peer-reviewed journal or conference proceedings; (ii) is the study set within the UK NHS; (iii) is the study conducted within the ED of an established hospital; and (iv) does the study use a computer simulation model of emergency patient flow? A study with positive responses on these four questions was then included in the final review. SM and JB completed this process independently and resolved any discrepancies that arose by consensus. Further to the electronic search, SM and JB reviewed the backward and forward citations of all studies included in the electronic search, to identify other potentially relevant studies.

Data extraction

For peer review only - http://bmjopen.bmj.com/site/about/guidelines.xhtml

An electronic data extraction form was created to retrieve information about a number of key aspects, including simulation methods, data sources, key assumptions, input and output data, conclusions drawn, and benefits of simulation outputs in practice. SM and JB independently recorded, collated, and extracted the necessary information. Any discrepancies were resolved by consensus.

RESULTS

We retrieved a total of 2,436 references from the eight databases: 437 from MEDLINE; 460 from EMBASE; 14 from COCHRANE; 253 from WEB OF SCIENCE; 65 from CINAHL; 1103 from INSPEC; 4 from MATHSCINET; and 100 from ACM DIGITAL LIBRARY. We removed 440 duplicate references, and then assessed the remaining 1,996 unique references by title and abstract screening. At this stage, we selected 159 of the 1,996 studies for full-text review. Nineteen of the 159 studies were included following full-text review. Two more studies were included from the backward and forward citation searching of the 19 studies. A total of 21 studies[10-14 21-36] were included in the final review. Four studies[37-40] were excluded from the final review because the models used by these studies are identical to the models reported in other already included studies.[13 30 33 36] Figure 1 shows a summary of the study selection process.

<Figure 1>

Table 1 summarises the included studies, outlining publication type, simulation purpose, ED details, and patient flow description. The first study[33] was published in 2000 and the most recent study[21] was published in 2013. The maximum number of studies (n=4) published in any single year was in 2006 and 2011. Nine of the 21 included studies (43%) were published in conference proceedings. The highest number of studies (n=7) was published in proceedings of the Winter Simulation Conference, the second highest (n=5) was in the Emergency Medicine Journal, and the third highest (n=3) was in the Journal of the Operational Research Society. More than two thirds of the studies (n=16; 76%) did not provide the name of the hospital studied. All 21 studies described the underlying purpose of simulation; and in many cases (n=16; 76%), this centred on service redesign. Surprisingly, 7 studies[12 13 21 25 31 34 36] (33%) did not provide any details about the ED being investigated, while 5 studies[10 21 23 26 30] (24%) did not provide patient flow diagram.

<Table 1>

For peer review only - http://bmjopen.bmj.com/site/about/guidelines.xhtml

Table 2 provides summary of simulation methods, including simulation type, key assumptions, and use of software. The types of simulation varied only between two methods (DES and SD). DES modelling was used in 19 studies (90%), while SD was used in 2 studies (10%). All but one study[25] either explicitly or implicitly justified the choice of underlying modelling method used. The majority of studies (n=18; 86%) used specific hospital models of ED patient flow. The reporting of modelling assumptions was poor overall. For example, as many as 12 studies[10 12 13 21 23-25 29 31 32 34 35] (57%) did not provide any details about simulation duration, warmup period, and run number. Only 5 studies[11 22 27 30 33] (24%) specified the number of simulation runs and 3 studies[27 30 36] (14%) specified the simulation warmup period. Simulation duration ranged between 24 hours[27 33] and 52 weeks.[26 30] Almost 50% (n=10) of the studies used Simul8 software (www.simul8.com) for running the model. Two studies[24 34] (10%) did not provide any details about the use of software.

<Table 2>

Table 3 provides detail of simulation inputs and outputs. The identified models were populated from three sources of data: primary (i.e. collected within the hospital being studied), secondary (i.e. collected in another setting), and expert opinion. One study[21] did not describe the source of data for any of the model inputs. Three studies[13 26 31] described the source of patient arrival rates, but not the sources of activity duration, activity progression, and use of resources. Eight studies (38%) stated explicitly that they used expert opinion to populate some of the model inputs. The proportion of studies that used primary data was reasonably high (Table 3). In particular, 95% (n=20) of the studies used primary data for patient arrival rates, 67% (n=14) for activity duration, 62% (n=13) for activity progression, and 52% (n=11) for resource inputs.

The most common changes considered in the simulation studies were ED patient flow (e.g. changes in the triage system for arriving patients[30]) and resource capacity planning (e.g. changes in the number of cubicles[30]). However, one third of the studies[11 13 21 24 26 27 31] (n=7) did not provide any details about the changes considered by the simulation. The majority of studies (n=19; 81%) considered patient waiting times (i.e. time from arrival to discharge, admission, or transfer) as the key outcome measure. In particular, 11 studies[10 11 13 21 22 25 27 29-31 34] considered patient waiting times alone, 7 studies[12 23 24 28 32 35 36] considered patient waiting times and resources used, and the other study[33] considered patient waiting times, resources used, and elective cancellations. Two other outcome measures considered were resources used[26] and bed occupancy.[14]

Only 12 studies (57%) reported some involvement of stakeholders in the simulation study, mainly when deciding the study questions or specifying the model structure. However, in the study conducted by Mould et al,[36] stakeholders were involved in deciding the study questions, specifying

For peer review only - http://bmjopen.bmj.com/site/about/guidelines.xhtml

the model structure, and implementing the model outputs. More than 80% (n=17) of the studies carried out some form of validation, mainly face and/or data-led validation. In face validation, project team members, potential users and other stakeholders subjectively compare model and real-life behaviours to judge whether the model and its results are reasonable at “face value”.[41] Data-led validation involves the comparing of model output with “real world” data and may also include a sensitivity analysis to determine the effect of varying the model’s inputs on its output performance.[42]

<Table 3>

Table 4 describes simulation results, summarising conclusions in terms of whether the changes considered (e.g. increase in staffing numbers) were supported by the simulation, whether the changes supported were implemented in practice (e.g. staffing increased), and barriers to conducting the simulation (e.g. data issues) and implementing the changes supported (e.g. poor clinician buy-in and credibility). Two thirds of the studies (n=14; 67%) provided some discussion on the usefulness of simulation for analysis of changes to the delivery of emergency care (Table 4): 6 studies supported the proposed changes, 1 study opposed the proposed changes, and 7 studies recommended differential changes. Only a small number of studies[12 34 36] (n=3; 14%) reported that the proposed changes supported by the simulations were implemented. For example, Maull et al[34] estimated the impact of introducing a ‘see and treat’ strategy to reduce patient waiting times in the ED. After implementation, the observed reduction in breaches of the 4-hour waiting time target closely mirrored the simulation model predictions.

We identified a broad range of challenges, including oversimplified assumptions[22 25 33 35] and model structure,[14 25] system complexity,[11 14 30 31 34] poor data quality,[12 25 29 34 36] high expectations,[24] short-timescale,[33] poor stakeholder engagement,[12] limited specialist analytical skills,[36] model runtime,[11 24] generalisability,[14 28] and impact of simulation;[36] 6 studies[10 21 23 26 27 32] (29%) did not describe any emergent issues.

<Table 4>

DISCUSSION

This review has shown that computer simulation has been used to analyse ED patient flow and resource capacity planning to the delivery of emergency care. The most common types of computer simulation used were DES (n=19; 90%) and SD (n=2; 10%). All but one study[25] provided either explicit or implicit justification for the choice of modelling method used. However, the use of computer simulation of patient flow within EDs in the UK does not appear to have increased in recent years as

For peer review only - http://bmjopen.bmj.com/site/about/guidelines.xhtml

may have been expected. This could be a reflection of the relatively limited availability of funding for research in this area compared to funding for health technology assessment. There are also a limited number of research groups with the analytical skills required to develop technically complex simulation models for the analysis of service redesign.

Identified studies varied in the style and quality of reporting; but assumptions used in the analyses were not always transparently reported. The opaque reporting of key assumptions prevents decision-makers from appraising the quality of evidence from simulation experiments. Although there is a set of guidelines for researchers of DES to follow when building models,[43] this has not been widely adopted yet. Most of the studies (n=19; 90%) considered patient waiting or throughput times as the main outcome measure. This is perhaps unsurprising since waiting time has been shown to be a key determinant of patient satisfaction and has been strongly prioritised through the 4-hour targets.[2] Some studies[13 21 26 31] did not provide enough information on how input parameters were selected and synthesised. A handful of studies used expert opinion to populate some of the model inputs, but none explicitly justified the reason for using expert opinion. It is important to have transparent criteria for using expert opinion since it can over or under estimate the model inputs. There are several methods for eliciting expert opinion as discussed by Grigore et al.[44]

Most models were intended to capture specific aspects of the emergency care process, but some authors have argued that understanding of patient flow requires study of the entire care process.[45] Conversely, others argue that it is sufficient to focus on the specific needs of the care process rather than modelling a large and complicated care process.[27] Most of the studies (n=18; 86%) used specific hospital models of ED patient flow. Interestingly, there seemed to be no standard hospital model of patient flow of emergency care process. One generic model was developed by the Department of Health in 2007 for use across all EDs.[12] This generic approach allows hospitals to benefit from simulation methodology with minimal costs and technical expertise, but there are challenges of using a generic national model for specific local use due to the local context of each NHS hospital including differences in physical space, the demographics of local patient populations, and so on.

Just over half of the studies in our review reported some involvement of stakeholders in the simulation study. Involving stakeholders is important since it helps to understand the problem better,[8 46] assess the simulation outputs fully,[47] and translate simulation outputs into policy.[15] Very few studies reported clear summaries of whether the changes considered were supported by the simulation and of whether the changes supported were implemented. Some studies drew attention to a number of challenges particularly associated with simulation conduct and implementation.

For peer review only - http://bmjopen.bmj.com/site/about/guidelines.xhtml

Brailsford[48] provided a helpful discussion on how to overcome the barriers such as methodological suitability, data crisis, and stakeholder issues.

Only 3 studies[12 34 36] (14%) reported on the implementation of the changes supported by the simulation outputs. This may show that the impact of computer simulation modelling within the field of UK's emergency care has been limited, though we do not know if any changes were implemented at a later date. We also do not know if any changes implemented led to any improvements in the process or outcomes of ED care. The systematic use of simulation modelling is not yet part of healthcare, whereas its use in other sectors like in manufacturing or airline industry is an integral part of the actual decision-making process.[49] Why is simulation yet to make the same impact in healthcare as in other industries? Lack of stakeholders' engagement has been argued as one of the main reasons for this.[8 46 48 49] To this end, Harper et al[46] discussed the basic components of successful implementation of simulation methods in healthcare. Absence of lucid guidelines about how to use simulation methods effectively in healthcare has been argued as another reason.[50] However, more recently in 2012, the ISPOR-SMDM Modeling Good Research Practices Task Force-4 laid out a set of guidelines about how to use DES method effectively in healthcare.[43] In line with a few others,[8 46 48 49] we also argue that if simulation is to make sustained impact in healthcare, the clinicians and decision-makers must cooperate across physical and organisational boundaries and come to understand how seemingly small changes in design of processes can improve patient care.

We systematically searched eight bibliographic databases to identify the included studies, however our study has some limitations. Firstly, we focused on the use of computer simulation methods in the context of patient flow within EDs under the jurisdiction of UK NHS only. Improving emergency care is a research priority for UK NHS.[1] In this review, we examined the current literature that analysed ED patient flow within the context of UK, and discussed how simulation can be better used as a tool to address this problem. It would be interesting to compare the identified methods with other jurisdictions, across Europe, in the USA and Australasia, but this was beyond the scope of this study. Besides, comparing studies from different jurisdictions and reaching consensus would be challenging since healthcare delivery is different in the UK. Nevertheless, computer simulation has been used to analyse and design ED overcrowding in other countries. In particular, DES models have been used to identify optimal ED flow patterns,[51] forecast ED overcrowding,[52 53] and evaluate staffing levels and changes in ED bed capacity.[54] Fletcher et al[12] cited a number of other international ED models which have different designs to English ED.

Secondly, we were not aware of any formal assessment checklist to estimate quality scores of the identified studies. The set of guidelines reported by the ISPOR-SMDM is not a quality assessment checklist for reviewers.[43] It is rather a set of recommended best practices for modelling teams to consider and embrace when building DES models. Furthermore, there is a good rationale

For peer review only - http://bmjopen.bmj.com/site/about/guidelines.xhtml

for a component-based approach, instead of using a quality score. For example, in the field of randomised controlled trials (RCTs), there is evidence that the use of quality scores and scales, especially of those with a numerical summary, is problematic and meaningless.[55] The current best practice for assessment of validity of RCTs requires assessing individual components of trial design, conduct and analysis (e.g. Cochrane risk of bias tool). We adopted a similar approach, whereby assessing the key methodological components of all included studies.

Thirdly, we neither verified whether any of the hospitals implemented the findings found from simulation experiments, nor do we know if any changes implemented led to any improvements. Typically, there is little opportunity to assess the impact of the simulation since publication emerges before the work is fully implemented in many healthcare studies.[56] Finally, we did not include Google Scholar in the database search list since it has a number of issues with its indexing and citation algorithm, although it is known to provide increased access to non peer-reviewed publications.[57] Anecdotal evidence suggests that NHS hospitals have used simulation modelling (and other methods) to improve patient flow through the ED. However, our review will not capture all of this work as it is not all reported in peer-reviewed academic publications. We used a key review paper[20] to select a wide range of databases covering the comprehensive sources of literature in computer science, operations management, and healthcare fields.

CONCLUSIONS

We found that computer simulation can provide a means to pre-test the likely impact of changes to the delivery of emergency care before implementation in a safe and efficient manner. In particular, it is used to identify the key relationships and bottlenecks in the process of ED care, test 'what-if' scenarios for service redesign, determine levels of uncertainty, provide visualisations, and forecast future performance. However, the evidence base is small and poorly developed, with many methodological and practical issues, including lack of awareness regarding system complexity, lack of good quality data, lack of persistent engagement of stakeholders in the modelling process, lack of in-house analytical skills, and lack of an implementation plan. Furthermore, the level of detail of reporting of the computer simulation methods differed in the style and quality of reporting; and in some instances, key aspects of the assumptions underpinning the analyses were not always reported explicitly and transparently.

This review is a useful source providing direction on why simulation needs to be better used as a tool for analysis of ED patient flow. Future studies should justify the choice of simulation modelling method explicitly, avoid making selective use of the available data, engage stakeholders in the modelling process and keep them on board continually, be transparent in the reporting of simulation inputs and outputs, and report on the implementation of changes supported by the

For peer review only - http://bmjopen.bmj.com/site/about/guidelines.xhtml

For peer review only

findings of simulation experiments. We recommend the adoption of reporting guidelines[43] by academic journals and conference proceedings, and more persistent exploitation of innovative models of engagement and knowledge mobilisation between academics and healthcare professionals such as the Researchers in Residence.[58] Further research is necessary to assess the quality of computer simulation models of ED patient flow across different countries and to establish the extent to which the simulation outputs have been translated into policy.

ACKNOWLEDGEMENTS

All the authors apart from CV are supported by the National Institute for Health Research (NIHR) Collaboration for Leadership in Applied Health Research and Care West (CLAHRC West) at University Hospitals Bristol National Health Service (NHS) Foundation Trust. The views expressed are those of the authors and not necessarily those of the NHS, the NIHR, or the Department of Health.

CONTRIBUTORS

SM and JB independently screened and appraised the relevant studies, and extracted the data from the included studies. CV, SM, JS, KN, JB, and AR designed the search strategy. AR, an information specialist, conducted the electronic search. SM drafted the manuscript in conjunction with CV. JD, WH, and CV revised the draft critically for intellectual content, while other authors commented on the draft. All authors read and approved the submitted manuscript.

FUNDING

NIHR

COMPETING INTERESTS

None

For peer review only

Table 1: Detail of the included studies

Study Name (Year)	Publication Type (Name)	Hospital Name	Simulation Purpose	ED Detail	Patient Flow Description
Anagnostou et al[21] (2013)	Conference proceedings (Winter Simulation Conference)	Unknown several hospitals in Greater London	Proof of concept	No detail	Textual; Activity list
Au-Yeung et al[22] (2006)	Conference proceedings (Modelling and Simulation)	Unknown hospital in North London	Service redesign	More detail	Flow chart; Textual
Baboolal et al[23] (2012)	Journal article (Emergency Medicine Journal)	University hospital of Wales	Service redesign	More detail	Textual
Bowers et al[24] (2009)	Journal article (Journal of Simulation)	Unknown hospital in Fife, Scotland	Service redesign	Some detail	Flow chart
Brailsford et al[10] (2004)	Journal article (Journal of Operational Research Society)	Nottingham city hospital & QMC in Nottingham	Service redesign	More detail	Textual
Coats et al[25] (2001)	Journal article (Emergency Medicine Journal)	Royal London hospital in Whitechapel, London	Service redesign	No detail	Flow chart
Codrington-Virtue et al[26] (2006)	Conference proceedings (Computer-Based Medical Systems)	Unknown hospital	Understand capacity	More detail	Textual
Codrington-Virtue et al[27] (2011)	Conference proceedings (Winter Simulation Conference)	Unknown hospital	Proof of concept	Some detail	Flow chart; Textual
Coughlan et al[28] (2011)	Journal article (Emergency Medicine Journal)	Unknown district general hospital in West London	Service redesign	Some detail	Flow chart; Textual
Davies et al[29] (2007)	Conference proceedings (Winter Simulation Conference)	Unknown hospital	Service redesign	More detail	Flow chart; Textual
Eatock et al[11] (2011)	Journal article (Journal of Health Org. and Management)	Hillingdon hospital in West London	Service redesign	More detail	Flow chart; Textual
Fletcher et al[12] (2007)	Journal article (Journal of Operational Research Society)	Unknown hospitals (n=10)	Service redesign	No detail	Flow chart; Textual
Gunal et al[13] (2009)	Journal article (Emergency Medicine Journal)	Unknown hospital	Understand behaviour	No detail	Flow chart; Textual
Gunal et al[30] (2006)	Conference proceedings (Winter Simulation Conference)	Unknown hospital	Service redesign	Some detail	Textual; Activity list
Hay et al[31] (2006)	Conference proceedings (Winter Simulation Conference)	Unknown hospitals (n=4)	Understand behaviour	No detail	Flow chart; Textual
Komashie et al[32] (2005)	Conference proceedings (Winter Simulation Conference)	Unknown hospital in London	Service redesign	More detail	Flow chart
Lane et al[33] (2000)	Journal article (Journal of Operational Research Society)	Unknown teaching hospital in London	Service redesign; Forecasting	More detail	Flow chart; Textual
Lattimer et al[14] (2004)	Journal article (Emergency Medicine Journal)	Nottingham city hospital & QMC in Nottingham	Service Redesign; Forecasting	Some detail	Flow chart
Maull et al[34] (2009)	Journal article (The Service Industries Journal)	Unknown hospital in South West of England	Service redesign; Forecasting	No detail	Flow chart
Meng et al[35] (2008)	Conference proceedings (Winter Simulation Conference)	Unknown hospital	Service redesign	More detail	Flow chart; Textual
Mould et al[36] (2013)	Journal article (Health Systems)	Unknown hospital in Fife, Scotland	Service redesign	No detail	Flow chart

QMC = Queen's Medical Centre

For peer review only

For peer review only - http://bmjopen.bmj.com/site/about/guidelines.xhtml

Table 2: Summary of simulation methods

Study Name	Simulation Type	Rationale for Simulation Type	Model Type	Simulation Duration	Warmup Period	Simulation Run	Simulation Software
Anagnostou et al[21]	DES[a]	Yes	Specific	Not reported	Not reported	Not reported	RePastSimphony
Au-Yeung et al[22]	DES[b]	Yes	Specific	Not reported	Not reported	10	Written in Java
Baboolal et al[23]	DES	Yes	Specific	Not reported	Not reported	Not reported	Simul8
Bowers et al[24]	DES	Yes	Specific	Not reported	Not reported	Not reported	Not reported
Brailsford et al[10]	DES[c]	Yes	Specific	Not reported	Not reported	Not reported	Simul8
Coats et al[25]	DES	No	Specific	Not reported	Not reported	Not reported	Simul8
Codrington-Virtue et al[26]	DES	Yes	Specific	52 weeks	Not reported	Not reported	Simul8
Codrington-Virtue et al[27]	DES	Yes	Specific	24 hours	24 hours	50	Simul8
Coughlan et al[28]	DES	Yes	Specific	3 weeks	Not reported	Not reported	Simul8
Davies et al[29]	DES	Yes	Specific	Not reported	Not reported	Not reported	Simul8
Eatock et al[11]	DES	Yes	Specific	3 weeks	Not reported	20	Simul8
Fletcher et al[12]	DES	Yes	Generic	Not reported	Not reported	Not reported	Simul8
Gunal et al[13]	DES[d]	Yes	Generic	Not reported	Not reported	Not reported	Micro Saint Sharp
Gunal et al[30]	DES[e]	Yes	Generic	52 weeks	0	50	Micro Saint Sharp
Hay et al[31]	DES	Yes	Specific	Not reported	Not reported	Not reported	Arena
Komashie et al[32]	DES	Yes	Specific	Not reported	Not reported	Not reported	Arena
Lane et al[33]	SD[f]	Yes	Specific	24 hours	Not reported	6	iThink
Lattimer et al[14]	SD[g]	Yes	Specific	52 weeks	Not reported	Not reported	Stella
Maull et al[34]	DES	Yes	Specific	Not reported	Not reported	Not reported	Not reported
Meng et al[35]	DES	Yes	Specific	Not reported	Not reported	Not reported	MedModel
Mould et al[36]	DES[h]	Yes	Specific	3 months	24 hours	Not reported	Simul8

DES = Discrete Event Simulation

SD = System Dynamics

[a] the authors used an agent based simulation approach to model the ambulance service, but modelled the ED through a DES. These two individual models were then linked together to form a hybrid emergency services model

[b] the authors used a Markovian queueing network, but computed the moments and densities of patient treatment time through a DES

[c] the authors used an SD model as part of a bigger picture, but modelled the ED through a DES

[d] the authors used their ED model elsewhere[37] to form a whole hospital DES model consisting of two other departments: inpatient and outpatient clinics

[e] the authors used their ED model elsewhere[38] to form a whole hospital DES model consisting of three other components: inpatient bed management, waiting list management, and outpatient clinics

[f] the authors used their ED model elsewhere[39] to explore the issues that arise when involving healthcare professionals in the process of model building

[g] the authors constructed the ED as a separate sub-model which was not detailed in the paper. However, we believe this ED sub-model[14] is identical to the ED model reported in another included study[10]

[h] the authors used their ED model elsewhere[40] to illustrate the role of care pathways to the redesign of healthcare systems

For peer review only - http://bmjopen.bmj.com/site/about/guidelines.xhtml

Table 3: Detail of simulation inputs and outputs

Study Name	Source of Arrival Rates	Source of Activity Duration	Source of Activity Progression	Source of Resources Use	Changes Considered	Outcomes Considered	Validation	Stakeholder Input
Anagnostou et al[21]	Not described	Not described	Not described	Not described	None	Waiting times	None	None
Au-Yeung et al[22]	Primary	Primary; Expert opinion	Primary; Expert opinion	Primary	ED patient flow	Waiting times	Data-led	Model specification
Baboolal et al[23]	Primary	Primary; Expert opinion	Primary	Primary	Resources	Waiting times*; Resources	Face Dark world model	Model specification
Bowers et al[24]	Primary	Primary	Primary	Not described	None	Resources*	Data-led Face	Model specification
Brailsford et al[10]	Primary	Secondary	Not described	Primary	ED patient flow; Arrival rates	Waiting times	Data-led Face	Study question; Model specification
Coats et al[25]	Primary	Primary	Not described	Not described	Shift patterns	Waiting times*	Data-led	None
Codrington-Virtue et al[26]	Primary	Not described	Not described	Not described	None	Resources	None	None
Codrington-Virtue et al[27]	Primary	Primary; Expert opinion	Primary	Primary; Expert opinion	None	Waiting times	Data-led	None
Coughlan et al[28]	Primary	Not described	Primary	Primary	Resources	Waiting times*; Resources	Data-led	None
Davies et al[29]	Primary	Primary	Primary	Primary	ED patient flow	Waiting times*	None	None
Eatock et al[11]	Primary	Primary; Expert opinion	Primary; Expert opinion	Primary	None	Waiting times*	Data-led	None
Fletcher et al[12]	Primary	Secondary; Expert opinion	Primary	Primary	ED patient flow; Resources; Demand	Waiting times*; Resources	Data-led Face	Study question; Model specification
Gunal et al[13]	Primary	Not described	Not described	Not described	None	Waiting times*	None	None
Gunal et al[30]	Primary	Primary	Primary	Not described	ED patient flow; Resources	Waiting times*	Data-led	Model specification
Hay et al[31]	Primary	Not described	Not described	Not described	None	Waiting times*	Data-led	None
Komashie et al[32]	Primary	Primary; Expert opinion	Primary; Expert opinion	Primary; Expert opinion	ED structure; Resources	Waiting times; Resources	Data-led Face	Study question; Model specification
Lane et al[33]	Primary	Primary; Expert opinion	Expert opinion	Primary	Resources; Demand	Waiting times; Resources; Elective cancellations	Data-led Face	Study question; Model specification
Lattimer et al[14]	Primary	Primary	Primary	Primary	ED Structure; Admission rates	Bed occupancy	Data-led Face	Study question; Model specification
Maull et al[34]	Primary	Primary; Expert opinion	Primary	Not described	ED patient flow	Waiting times*	Data-led	Result implementation
Meng et al[35]	Primary	Primary	Not described	Not described	ED structure; Resources	Waiting times; Resources	Data-led Face	Study question; Model specification
Mould et al[36]	Primary	Primary	Primary	Not described	Resources	Waiting times*; Resources	Data-led	Study question; Model specification; Result implementation

* these studies used 4-hour target breach as part of their waiting time considerations

For peer review only - http://bmjopen.bmj.com/site/about/guidelines.xhtml

Table 4: Summary of simulation results

Study Name	Conclusions	Conclusions Detail	Reported the Changes Implemented?	Result Implementation	Barriers
Anagnostou et al[21]	None	None	No	N/A	None
Au-Yeung et al[22]	Supported the changes considered	Prioritisation of treatment for patient with minor problems over major problems could lead to improved outcome	No	N/A	Simplified assumptions
Baboolal et al[23]	Supported the changes considered	A change in staffing levels could lead to substantial cost savings and reduce the 4-hour breaches	No	N/A	None
Bowers et al[24]	None	None	No	N/A	Model runtime; High expectancy
Brailsford et al[10]	Opposed the changes considered	Streaming of patients by triage category was not an efficient use of clinical resources	No	N/A	None
Coats et al[25]	Supported the changes considered	Shift pattern that best matches patient arrivals would give shorter waiting times	No	N/A	Simplified model structure and assumptions; Poor data quality
Codrington-Virtue et al[26]	None	None	No	N/A	None
Codrington-Virtue et al[27]	None	None	No	N/A	None
Coughlan et al[28]	Proposed differential changes	Adding an emergency nurse practitioner would not reduce the waiting times. Resource reallocation would improve throughput times	No	N/A	Generalisability
Davies et al[29]	Supported the changes considered	The separation of see and treat would be beneficial	No	N/A	Poor data quality
Eatock et al[11]	None	None	No	N/A	System complexity; Model runtime
Fletcher et al[12]	Proposed differential changes	Deflecting demand away from A&E would lead to improvement around waiting for beds, specialists, and assessment processes	Yes	Unknown as other interventions were introduced in parallel	Poor data quality; Poor stakeholder engagement
Gunal et al[13]	None	None	No	N/A	Explaining the causes of change in performance
Gunal et al[30]	Proposed differential changes	More senior doctors, less x-ray requisitions, and more cubicles would reduce waiting times	No	N/A	Modelling multitasking behaviour of staff
Hay et al[31]	None	None	No	N/A	System complexity
Komashie et al[32]	Proposed differential changes	Adding a nurse or doctor to minors would reduce the waiting times by 28%. Increasing the cubicles/beds would make smaller change	No	N/A	None
Lane et al[33]	Proposed differential changes	Changing bed numbers led to no noticeable change in waiting times but a substantial difference to elective cancelations	No	N/A	Short-timescale; Simplified assumptions
Lattimer et al[14]	Proposed differential changes	System would not be able to cope with increasing demand from scenario 1[a], but scenario 2[b], 3[c], and 4[d] could improve this	No	N/A	Simplified model structure; System complexity; Generalisability
Maull et al[34]	Supported the changes considered	See and treat reduced the 4-hour breaches from 13.2% to 3.4%	Yes	Marked reduction in no. of breaches from 13.2% to 1.4%. No. of patients waiting less than 1-hour increased from 12% to 23%. No. of patients with major problems waiting between 3 and 4 hours increased	Poor data availability and quality; System complexity
Meng et al[35]	Proposed differential changes	Reduced times to see a consultant would reduce the waiting times. Access to 24-hour x-ray would reduce the waiting times too	No	N/A	Simplified assumptions
Mould et al[36]	Supported the changes considered	A new staff roster would reduce the waiting times	Yes	Mean time for minor problems dropped from 100 to 94 mins, for major problems it dropped from 200 to 195 mins. Mean time for minor problems fell by 16 mins	Poor data quality; Limited analytical skills; Impact of simulation

For peer review only - http://bmjopen.bmj.com/site/about/guidelines.xhtml

				after adjusting other factors	

[a] 5-year model run assuming 4% year on year growth in emergency admissions and 3% year on year growth in GP referral for planned admissions
[b] impact of increase in demand for front door services
[c] reducing emergency admissions of patients with respiratory or coronary problems, ill-defined conditions, and over 65 years
[d] effects of earlier discharge of patients admitted as emergencies and subsequently discharged to nursing or residential homes

For peer review only

For peer review only - http://bmjopen.bmj.com/site/about/guidelines.xhtml

REFERENCES

1. Banerjee A, Mbamalu D, Hinchley G. The impact of process re-engineering on patient throughput in emergency departments in the UK. *Int J Emerg Med* 2008;1(3):189-92. doi: 10.1007/s12245-008-0055-x
2. Hemaya SA, Locker TE. How accurate are predicted waiting times, determined upon a patient's arrival in the Emergency Department? *Emergency Medicine Journal* 2012;29(4):316-18.
3. Trzeciak S, Rivers EP. Emergency department overcrowding in the United States: an emerging threat to patient safety and public health. *Emerg Med J* 2003;20(5):402-5.
4. Miro O, Antonio MT, Jimenez S, et al. Decreased health care quality associated with emergency department overcrowding. *Eur J Emerg Med* 1999;6(2):105-7.
5. Trout A, Magnusson AR, Hedges JR. Patient satisfaction investigations and the emergency department: what does the literature say? *Acad Emerg Med* 2000;7(6):695-709.
6. Fatovich DM, Hirsch RL. Entry overload, emergency department overcrowding, and ambulance bypass. *Emerg Med J* 2003;20(5):406-9.
7. Blunt I. Focus on: A&E attendances. Why are patients waiting longer? Quality Watch, The Health Foundation and Nuffield Trust. 2014 [31 August 2016]. Available from: http://www.nuffieldtrust.org.uk/sites/files/nuffield/publication/140724_focus_on_ae_attendances.pdf.
8. Carrus B, Corbett S, Khandelwal D. A hospital-wide strategy for fixing emergency-department overcrowding. *McKinsey Quarterly* 2010:1-12.
9. de Koning H, Verver JP, van den Heuvel J, et al. Lean six sigma in healthcare. *J Healthc Qual* 2006;28(2):4-11.
10. Brailsford S, Lattimer V, Tarnaras P, et al. Emergency and on-demand health care: modelling a large complex system. *J Oper Res Soc* 2004;55(1):34-42. doi: 10.1057/palgrave.jors.2601667
11. Eatock J, Clarke M, Picton C, et al. Meeting the four-hour deadline in an A&E department. *Journal of health organization and management* 2011;25(6):606-24.
12. Fletcher A, Halsall D, Huxham S, et al. The DH Accident and Emergency Department model: a national generic model used locally. *J Oper Res Soc* 2007;58(12):1554-62. doi: DOI 10.1057/palgrave.jors.2602344
13. Günal MM, Pidd M. Understanding target-driven action in emergency department performance using simulation. *Emergency Medicine Journal* 2009;26(10):724-27. doi: 10.1136/emj.2008.066969
14. Lattimer V, Brailsford S, Turnbull J, et al. Reviewing emergency care systems I: insights from system dynamics modelling. *Emergency Medicine Journal* 2004;21(6):685-91. doi: 10.1136/emj.2002.003673
15. Sobolev B, Kuramoto L. Policy analysis using patient flow simulations: Conceptual framework and study design. *Clin Invest Med* 2005;28(6):359-63.
16. Pitt M, Monks T, Crowe S, et al. Systems modelling and simulation in health service design, delivery and decision making. *BMJ Qual Saf* 2016;25(1):38-45. doi: 10.1136/bmjqs-2015-004430
17. Davies R, Roderick P, Raftery J. The evaluation of disease prevention and treatment using simulation models. *Eur J Oper Res* 2003;150(1):53-66. doi: 10.1016/S0377-2217(02)00783-X
18. Brailsford S, Hilton N. A comparison of discrete event simulation and system dynamics for modelling health care systems. 2001
19. Hutzschenreuter A, Bosman P, Blonk-Altena I, et al. Agent-based patient admission scheduling in hospitals. Proceedings of the 7th international joint conference on Autonomous agents and multiagent systems: industrial track; 2008. International Foundation for Autonomous Agents and Multiagent Systems.
20. Sobolev BG, Sanchez V, Vasilakis C. Systematic review of the use of computer simulation modeling of patient flow in surgical care. *J Med Syst* 2011;35(1):1-16. doi: 10.1007/s10916-009-9336-z
21. Anagnostou A, Nouman A, Taylor SJE. Distributed Hybrid Agent-Based Discrete Event Emergency Medical Services Simulation. *Wint Simul C Proc* 2013:1625-36.

For peer review only - http://bmjopen.bmj.com/site/about/guidelines.xhtml

22. Au-Yeung SWM, Harrison PG, Knottenbelt WJ. A queueing network model of patient flow in an accident and emergency department. *Modelling and Simulation 2006* 2006:60-67.
23. Baboolal K, Griffiths JD, Knight VA, et al. How efficient can an emergency unit be? A perfect world model. *Emergency Medicine Journal* 2012;29(12):972-77.
24. Bowers J, Ghattas M, Mould G. Success and failure in the simulation of an accident and emergency department. *Journal of Simulation* 2009;3(3):171-78.
25. Coats TJ, Michalis S. Mathematical modelling of patient flow through an accident and emergency department. *Emergency Medicine Journal* 2001;18(3):190-92. doi: DOI 10.1136/emj.18.3.190
26. Codrington-Virtue A, Chaussalet T, Millard P, et al. A system for patient management based discrete-event simulation and hierarchical clustering. *Comp Med Sy* 2006:800-04. doi: Doi 10.1109/Cbms.2006.31
27. Codrington-Virtue A, Kelly J, Chaussalet T. Using Simplified Discrete-Event Simulation Models for Health Care Applications. *Proceedings of the 2011 Winter Simulation Conference (WSC)* 2011:1154-65.
28. Coughlan J, Eatock J, Patel N. Simulating the use of re-prioritisation as a wait-reduction strategy in an emergency department. *Emergency Medicine Journal* 2011;28(12):1013-18. doi: 10.1136/emj.2010.100255
29. Davies R. “See and Treat” or “See” and “Treat” in an emergency department. 2007 Winter Simulation Conference; 2007. IEEE.
30. Günal MM, Pidd M. Understanding accident and emergency department performance using simulation. *Proceedings of the 2006 Winter Simulation Conference, Vols 1-5* 2006:446-52. doi: Doi 10.1109/Wsc.2006.323114
31. Hay A, Valentin E, Bijlsma R. Modeling emergency care in hospitals: a paradox-the patient should not drive the process. Proceedings of the 2006 Winter Simulation Conference; 2006. IEEE.
32. Komashie A, Mousavi A. Modeling emergency departments using discrete event simulation techniques. Proceedings of the 37th conference on Winter simulation; 2005. Winter Simulation Conference.
33. Lane DC, Monefeldt C, Rosenhead JV. Looking in the wrong place for healthcare improvements: A system dynamics study of an accident and emergency department. *J Oper Res Soc* 2000;51(5):518-31. doi: Doi 10.2307/254183
34. Maull RS, Smart P, Harris A, et al. An evaluation of ‘fast track’in A&E: a discrete event simulation approach. *The Service Industries Journal* 2009;29(7):923-41.
35. Meng LY, Spedding T. Modelling Patient Arrivals When Simulating an Accident and Emergency Unit. *2008 Winter Simulation Conference, Vols 1-5* 2008:1509-15.
36. Mould G, Bowers J, Dewar C, et al. Assessing the impact of systems modeling in the redesign of an Emergency Department. *Health Systems* 2013;2(1):3-10.
37. Günal MM, Pidd M. Interconnected DES models of emergency, outpatient, and inpatient departments of a hospital. 2007 Winter simulation conference; 2007. IEEE.
38. Günal MM, Pidd M. DGHPSIM: Generic simulation of hospital performance. *ACM Transactions on Modeling and Computer Simulation (TOMACS)* 2011;21(4):23.
39. Lane DC, Monefeldt C, Husemann E. Client involvement in simulation model building: hints and insights from a case study in a London hospital. *Health Care Manag Sci* 2003;6(2):105-16.
40. Mould G, Bowers J. Roles of pathway-based models and their contribution to the redesign of health-care systems. *International Journal of Care Pathways* 2011;15(3):90-97.
41. Balci O. Validation, verification, and testing techniques throughout the life cycle of a simulation study. *Annals of operations research* 1994;53(1):121-73.
42. Robinson S. Simulation: the practice of model development and use. *Hoboken, NJ: John Wiley & Sons* 2004
43. Karnon J, Stahl J, Brennan A, et al. Modeling using discrete event simulation: a report of the ISPOR-SMDM Modeling Good Research Practices Task Force-4. *Value Health* 2012;15(6):821-7. doi: 10.1016/j.jval.2012.04.013

For peer review only

44. Grigore B, Peters J, Hyde C, et al. A comparison of two methods for expert elicitation in health technology assessments. *BMC Med Res Methodol* 2016;16:85. doi: 10.1186/s12874-016-0186-3
45. Haraden C, Resar R. Patient flow in hospitals: understanding and controlling it better. *Front Health Serv Manage* 2004;20(4):3-15.
46. Harper PR, Pitt M. On the challenges of healthcare modelling and a proposed project life cycle for successful implementation. *J Oper Res Soc* 2004;55(6):657-61.
47. Eldabi T, Irani Z, Paul RJ. A proposed approach for modelling health-care systems for understanding. *J Manag Med* 2002;16(2-3):170-87.
48. Brailsford S. Overcoming the barriers to implementation of operations research simulation models in healthcare. *Clin Invest Med* 2005;28(6):312-5.
49. Eldabi T, Paul R, Young T. Simulation modelling in healthcare: reviewing legacies and investigating futures. *J Oper Res Soc* 2007;58(2):262-70.
50. Jun GT, Ward J, Morris Z, et al. Health care process modelling: which method when? *International Journal for Quality in Health Care* 2009;21(3):214-24.
51. Hung GR, Whitehouse SR, O'Neill C, et al. Computer modeling of patient flow in a pediatric emergency department using discrete event simulation. *Pediatr Emerg Care* 2007;23(1):5-10. doi: 10.1097/PEC.0b013e31802c611e
52. Connelly LG, Bair AE. Discrete event simulation of emergency department activity: a platform for system-level operations research. *Acad Emerg Med* 2004;11(11):1177-85. doi: 10.1197/j.aem.2004.08.021
53. Hoot NR, LeBlanc LJ, Jones I, et al. Forecasting emergency department crowding: a discrete event simulation. *Ann Emerg Med* 2008;52(2):116-25. doi: 10.1016/j.annemergmed.2007.12.011
54. Khare RK, Powell ES, Reinhardt G, et al. Adding more beds to the emergency department or reducing admitted patient boarding times: which has a more significant influence on emergency department congestion? *Annals of emergency medicine* 2009;53(5):575-85. e2.
55. Juni P, Witschi A, Bloch R, et al. The hazards of scoring the quality of clinical trials for meta-analysis. *JAMA* 1999;282(11):1054-60.
56. Fone D, Hollinghurst S, Temple M, et al. Systematic review of the use and value of computer simulation modelling in population health and health care delivery. *J Public Health Med* 2003;25(4):325-35.
57. Shultz M. Comparing test searches in PubMed and Google Scholar. *Journal of the Medical Library Association: JMLA* 2007;95(4):442.
58. Marshall M, Pagel C, French C, et al. Moving improvement research closer to practice: the Researcher-in-Residence model. *BMJ Qual Saf* 2014;23(10):801-5. doi: 10.1136/bmjqs-2013-002779

For peer review only

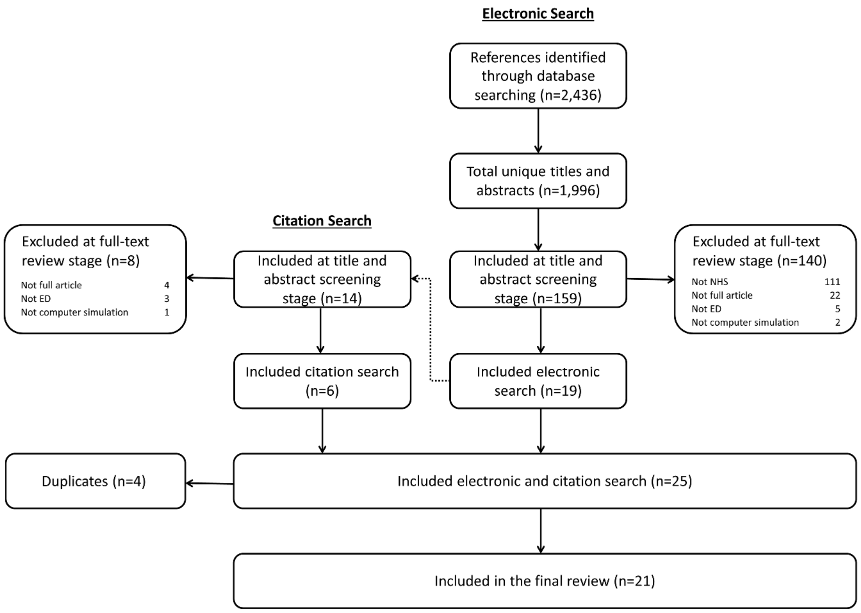

Figure 1 Flowchart of the study identification and inclusion process.

400x400mm (300 x 300 DPI)

APPENDIX 1

MEDLINE search strategy

----

1 exp Computer Simulation/ (175146)

2 models, statistical/ (75054)

3 ((computer* or distributed or hybrid) adj6 (model* or simulation*)).mp. (184347)

4 ((flow or simulation) adj6 model$).mp. (41697)

5 (system adj3 model$).mp. (50666)

6 (discrete adj2 event).mp. (783)

7 (monte adj2 carlo).mp. (40800)

8 markov.mp. (17983)

9 (statechart$ or stochastic or system dynamics).mp. (32076)

10 (agent adj2 based).mp. (3106)

11 or/1-10 (391259)

12 workflows/ or crowding/ or exp waiting lists/ (13340)

13 time to treatment/ or triage/ or patient admission/ or length of stay/ (92230)

14 exp critical pathways/ or exp "Process Assessment (Health care)"/ (8528)

15 exp patient care planning/ or exp patient care/ or exp "continuity of patient care"/ (679418)

16 ((clinical or critical or patient*) adj2 (path or paths or pathways)).tw. (5722)

17 (patient$ adj3 (flow or flows or flowing or throughput)).tw. (11371)

18 (care adj3 (step or steps or stage$)).tw. (2424)

19 (process adj2 care).tw. (4165)

20 (workflow* or crowding* or waiting list* or waiting time*).tw. (31590)

21 or/12-20 (741748)

22 emergency medical services/ or emergency medical service communication systems/ or emergency service, hospital/ or exp ambulances/ (86523)

23 ((emergency or emergencies or unplanned or unscheduled) adj3 (care or admission$ or department$ or ward or wards or service$ or unit or units or room or rooms)).tw. (95179)

24 (A&E or "accident and emergency").tw. (18002)

25 (minor injur* adj2 (unit* or servic* or ward* or hospital* or department*)).ti,ab. (137)

26 22 or 23 or 24 or 25 (154509)

27 11 and 21 and 26 (437)

For peer review only

PRISMA 2009 Checklist

Section/topic	#	Checklist item	Reported on page #
**TITLE**			
Title	1	Identify the report as a systematic review, meta-analysis, or both.	1
**ABSTRACT**			
Structured summary	2	Provide a structured summary including, as applicable: background; objectives; data sources; study eligibility criteria, participants, and interventions; study appraisal and synthesis methods; results; limitations; conclusions and implications of key findings; systematic review registration number.	2
**INTRODUCTION**			
Rationale	3	Describe the rationale for the review in the context of what is already known.	5
Objectives	4	Provide an explicit statement of questions being addressed with reference to participants, interventions, comparisons, outcomes, and study design (PICOS).	5-6
**METHODS**			
Protocol and registration	5	Indicate if a review protocol exists, if and where it can be accessed (e.g., Web address), and, if available, provide registration information including registration number.	6
Eligibility criteria	6	Specify study characteristics (e.g., PICOS, length of follow-up) and report characteristics (e.g., years considered, language, publication status) used as criteria for eligibility, giving rationale.	6
Information sources	7	Describe all information sources (e.g., databases with dates of coverage, contact with study authors to identify additional studies) in the search and date last searched.	6
Search	8	Present full electronic search strategy for at least one database, including any limits used, such that it could be repeated.	Appendix 1
Study selection	9	State the process for selecting studies (i.e., screening, eligibility, included in systematic review, and, if applicable, included in the meta-analysis).	6-7
Data collection process	10	Describe method of data extraction from reports (e.g., piloted forms, independently, in duplicate) and any processes for obtaining and confirming data from investigators.	7
Data items	11	List and define all variables for which data were sought (e.g., PICOS, funding sources) and any assumptions and simplifications made.	7
Risk of bias in individual studies	12	Describe methods used for assessing risk of bias of individual studies (including specification of whether this was done at the study or outcome level), and how this information is to be used in any data synthesis.	N/A
Summary measures	13	State the principal summary measures (e.g., risk ratio, difference in means).	N/A
Synthesis of results	14	Describe the methods of handling data and combining results of studies, if done, including measures of consistency (e.g., I^2 for each meta-analysis.	N/A

For peer review only - http://bmjopen.bmj.com/site/about/guidelines.xhtml
For peer review only

PRISMA 2009 Checklist

Section/topic	#	Checklist item	Reported on page #
Risk of bias across studies	15	Specify any assessment of risk of bias that may affect the cumulative evidence (e.g., publication bias, selective reporting within studies).	N/A
Additional analyses	16	Describe methods of additional analyses (e.g., sensitivity or subgroup analyses, meta-regression), if done, indicating which were pre-specified.	N/A
**RESULTS**			
Study selection	17	Give numbers of studies screened, assessed for eligibility, and included in the review, with reasons for exclusions at each stage, ideally with a flow diagram.	7-8 Fig. 1
Study characteristics	18	For each study, present characteristics for which data were extracted (e.g., study size, PICOS, follow-up period) and provide the citations.	8
Risk of bias within studies	19	Present data on risk of bias of each study and, if available, any outcome level assessment (see item 12).	N/A
Results of individual studies	20	For all outcomes considered (benefits or harms), present, for each study: (a) simple summary data for each intervention group (b) effect estimates and confidence intervals, ideally with a forest plot.	Tables 1-4
Synthesis of results	21	Present results of each meta-analysis done, including confidence intervals and measures of consistency.	N/A
Risk of bias across studies	22	Present results of any assessment of risk of bias across studies (see Item 15).	N/A
Additional analysis	23	Give results of additional analyses, if done (e.g., sensitivity or subgroup analyses, meta-regression [see Item 16]).	N/A
**DISCUSSION**			
Summary of evidence	24	Summarize the main findings including the strength of evidence for each main outcome; consider their relevance to key groups (e.g., healthcare providers, users, and policy makers).	10-11
Limitations	25	Discuss limitations at study and outcome level (e.g., risk of bias), and at review-level (e.g., incomplete retrieval of identified research, reporting bias).	12
Conclusions	26	Provide a general interpretation of the results in the context of other evidence, and implications for future research.	12-13
**FUNDING**			
Funding	27	Describe sources of funding for the systematic review and other support (e.g., supply of data); role of funders for the systematic review.	14

From: Moher D, Liberati A, Tetzlaff J, Altman DG, The PRISMA Group (2009). Preferred Reporting Items for Systematic Reviews and Meta-Analyses: The PRISMA Statement. PLoS Med 6(7): e1000097. doi:10.1371/journal.pmed1000097

For more information, visit: www.prisma-statement.org.

For peer review only - http://bmjopen.bmj.com/site/about/guidelines.xhtml
